# Process evaluation of the healthy primary School of the Future: the key learning points

**DOI:** 10.1186/s12889-019-6947-2

**Published:** 2019-06-06

**Authors:** N. H. M. Bartelink, P. van Assema, M. W. J. Jansen, H. H. C. M. Savelberg, G. F. Moore, J. Hawkins, S. P. J. Kremers

**Affiliations:** 1 Department of Health Promotion, Care and Public Health Research Institute (CAPHRI), Maastricht University, P.O. Box 616, 6200 MD Maastricht, The Netherlands; 2 Department of Health Promotion, School of Nutrition and Translational Research in Metabolism (NUTRIM), Maastricht University, P.O. Box 616, 6200 MD Maastricht, The Netherlands; 3 Public Health Services, Academic Collaborative Centre for Public Health Limburg, P.O. Box 33, 6400 AA Heerlen, The Netherlands; 4 Department of Health Services Research, CAPHRI, Maastricht University, P.O. Box 616, 6200 MD Maastricht, The Netherlands; 5 Department of Nutrition and Movement Sciences, NUTRIM, Maastricht University, P.O. Box 616, 6200 MD Maastricht, The Netherlands; 6 Centre for the Development and Evaluation of Complex Interventions for Public Health Improvement (DECIPHer), School of Social Sciences, Cardiff University, 1-3 Museum Place, Cardiff, CF10 3BD Wales

**Keywords:** Action research, Complex systems, Context, Implementation, Mixed methods, School health promotion

## Abstract

**Background:**

While schools have potential to contribute to children’s health and healthy behaviour, embedding health promotion within complex school systems is challenging. The ‘Healthy Primary School of the Future’ (HPSF) is an initiative that aims to integrate health and well-being into school systems. Central to HPSF are two top-down changes that are hypothesized as being positively disruptive to the Dutch school system: daily free healthy lunches and structured physical activity sessions. These changes are expected to create momentum for bottom-up processes leading to additional health-promoting changes. Using a programme theory, this paper explores the processes through which HPSF and the school context adapt to one another. The aim is to generate and share knowledge and experiences on how to implement changes in the complex school system to integrate school health promotion.

**Methods:**

The current study involved a mixed methods process evaluation with a contextual action-oriented research approach. The processes of change were investigated in four Dutch primary schools during the development year (2014–2015) and the first two years of implementation (2015–2017) of HPSF. The schools (each with 15–26 teachers and 233–389 children) were in low socio-economic status areas. Measurements included interviews, questionnaires, observations, and analysis of minutes of meetings.

**Results:**

Top-down advice, combined with bottom-up involvement and external practical support were key facilitators in embedding HPSF within the schools’ contexts. Sufficient coordination and communication at the school level, team cohesion, and feedback loops enhanced implementation of the changes. Implementation of the healthy lunch appeared to be disruptive and create momentum for additional health-promoting changes.

**Conclusions:**

Initiating highly visible positive disruptions to improve school health can act as a catalyst for wider school health promotion efforts. Conditions to create a positive disruption are enough time, and sufficient bottom-up involvement, external support, team cohesion and coordination. The focus should be on each specific school, as each school has their own starting point and process of change.

**Trial registration:**

The study was retrospectively registered in the ClinicalTrials.gov database on 14 June 2016 (NCT02800616).

**Electronic supplementary material:**

The online version of this article (10.1186/s12889-019-6947-2) contains supplementary material, which is available to authorized users.

## Background

The school setting has the potential to influence children’s health and well-being, in part by supporting the adoption of healthy behaviours [1–3]. Establishing healthy behaviours at an early age may help to improve children’s health and educational achievements; both may lead to improved health later in life and closing the equity gaps in both health and academic achievement [4, 5]. However, school health promotion is globally often characterised by relatively low priority, fragmentation, and a lack of coordination [6, 7]. The Health Promoting School framework as defined by the World Health Organisation aims for a whole-school approach, and focuses on reorienting school systems toward health promotion through embedding health and well-being in the curriculum, creating healthy social and physical environments and engaging with parents and the wider community [8]. This concept has shown promise, though several studies (including the Netherlands) indicate that effects are often hampered by underestimation of the challenges associated with implementing meaningful whole-system changes [9–12].

Challenges associated with changing school systems vary between schools: every school has its own dynamics, shaped by a large number of interacting elements and ever-changing agents within it [10, 11, 13]. Schools can thus be conceptualised as complex systems. Key to this conceptualisation is an understanding of the non-linearity of systems and the ways in which feedback impacts overall system behaviours and adaptations over time [14]. An intervention can be seen as an attempt to positively disrupt the prior functioning of a system [15, 16]. Moreover, complexity goes beyond the school gates, as school is only one of a diversity of microsystems which interact to shape child development and wellbeing. Changes in children’s home setting and neighbourhood, other microsystems with which children interact, also interact with the impact of changes at school [17].

A Dutch initiative based on the Health Promoting School framework, and informed by a systems approach, is the ‘Healthy Primary School of the Future’ (HPSF). This initiative, with a focus on healthy nutrition and physical activity (PA), aims to improve children’s health and well-being by enhancing health promotion throughout the whole school system, with the aim of contributing to fostering a healthier future generation [18, 19]. Central to this HPSF-concept is the top-down initiation of two changes, a free healthy lunch each day and daily structured PA sessions. While in other national school systems these may represent usual practice, the two changes are hypothesized as being positively disruptive to the Dutch school system. In the Netherlands, children eat their lunch at home or bring lunch to eat at school; PA is restricted to one or two physical education classes a week and some free playtime during (lunch) breaks. Contextualisation of the two changes is supported by bottom-up involvement of teachers and parents. The changes aim to facilitate the conditions within the school context for healthy dietary and PA behaviours and to create momentum for more bottom-up processes that lead to additional health-promoting (HP) changes.

To better understand implementation processes [12], we conducted a process evaluation. In line with recent debates in this research area [13, 20–22], the focus in this process evaluation was not on the fidelity of intervention components in purely compositional terms, but on adaptation of the intervention and system to one another, and factors crucial for sustained change [12, 23]. The aim of the current study was to generate knowledge and experiences on how to implement changes in the complex school system to integrate school health promotion and to share key learning points. Specifically, the study explored the processes through which HPSF and the school context adapted to one another during the development year (academic year 2014/15) and the first two years of implementation (2015/17) in four schools. Three main research questions were formulated: 1) *What was the pre-existing context of the four schools prior to the introduction of HPSF?,* 2) *How was HPSF developed and implemented and how did it interact with the context of the four schools?,* and 3) *After two years, to what extent was HPSF integrated and did the context of the four schools change?*

## Methods

### Study design

This process evaluation is part of an overall study that investigates HPSF using a quasi-experimental study design [19]. The overall study includes four intervention schools and four control schools. The process evaluation reported here focuses on the four intervention schools, using mixed methods (Table 1). Data were collected during three years (2014–2017) in four intervention schools. Ethical approval was obtained from the Medical Ethics Committee Zuyderland located in Heerlen (the Netherlands). All four schools started implementation of HPSF in November 2015. Funding for implementation is provided until the end of 2019. However, the four schools have committed to continued implementation after 2019 and to making the changes sustainable in their school.

**Table 1:** Research questions and used methods

RQ and sub-RQ		Concepts and variables	Methods
RQ1: What was the pre-existing context of the four schools prior to the introduction of HPSF?		The school context: • HP practices of teachers/parents • HP elements in school • Dominant organisational issues • Perceived barriers for HPSF • Characteristics of student population	- Interviews - Minutes - Observations - Practices_Q - Barrier_Q - Open questions in Barrier_Q
RQ2: How was HPSF developed and implemented and how did it interact with the context of the four schools?	2.1: How were the two top-down changes developed and implemented in the four schools?	Two top-down changes: • Daily healthy lunch • Structured PA sessions	- Interviews - Minutes - Observations
	2.2: To which additional health promoting changes did the two top-down changes lead to in the four schools?	Changes in HP elements in school: • School routine • Policy • Education • Environment	- Interviews - Minutes - Observations
	2.3: Which (potential) barriers for HPSF were perceived by the implementers in the four schools, and how did they change during the first two years of implementation?	Perceived (potential) barriers for HPSF: • Innovation-related • Implementers-related • Organisation-related • Socio-political context-related	- Barrier_Q
	2.4: Which factors influenced the development and implementation of HPSF in the four schools during the first two years of implementation?	Development and implementation process of HPSF: • Coordination • Team cohesion • Bottom-up involvement • External support • Momentum	- Interviews - Minutes - Observations - Open questions in Barrier_Q
RQ3: After two years, to what extent was HPSF integrated and did the context of the four schools change?	3.1: What impacts did HPSF give rise to in the four schools after the first two years of implementation?	Changes in the school context: • HP elements in school • HP practices of teachers/parents • Characteristics of student population	- Interviews - Minutes - Observations - Practices_Q
	3.2: To what extent was HPSF seen as being fully integrated into the everyday functioning of the school after the first two years of implementation?	Perceived feelings of integration	- Interviews

Abbreviations: Barrier_Q = barrier questionnaire; Practices_Q = practices questionnaire; RQ = research question

### Programme theory

**Fig. 1:**
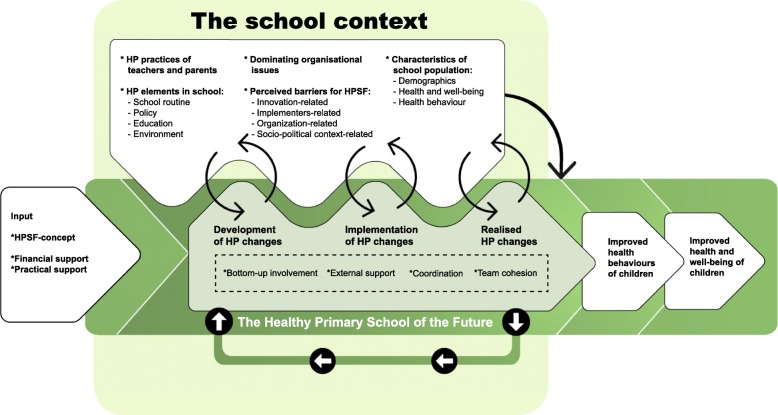
Programme theory

This study uses a contextual action-oriented research approach (CARA) [23]. CARA focuses on contextual differences, use of monitoring and inducing feedback loops to support and evaluate the processes of change in each school. Based on the principles of CARA and complex systems thinking, we developed a programme theory on the hypothesized processes of how HPSF integrates into the school context (Fig. 1). The HPSF-concept and the pre-arranged financial and practical support for its implementation, aim to act as an ‘event’ that positively disrupts the pre-existing dynamics in the school context [13, 15]. The context within and across schools acts as the starting point of HPSF [21]. Therefore, understanding relevant aspects of the pre-existing school context is required, such as HP practices of teachers and parents [10], HP elements in school (school routine, policy, education, and environment) [8], dominating organisational issues (e.g., staff turnover) [24], barriers for HPSF related to innovation, implementers, organisation, and socio-political context [25], and characteristics of the school population (demographics, health behaviours, health and well-being) [24]. The introduction of HPSF into the school context initiates the HPSF process of development, implementation, and integration [26]. Based on existing implementation literature it was hypothesized that coordination, team cohesion, bottom-up involvement, and external support would improve this process [9, 27–29]. During the process, feedback loops will develop in two directions [14]: on the one hand, the school context is expected to impact the HP change process, on the other hand, the context may respond to HP changes, which may result in a new way of working in the school context [10, 25]. Feedback loops may be positive, thereby amplifying the changes, or negative, thereby counteracting the changes [10, 14]. During this complex process of change, the system tries to find a new balance: it tends to self-organise to a new state of stability, either by pushing the change out of the system or by integrating the change into the system [30]. A key assumption of our programme theory concerns non-linearity in the cause-effect relationship, which means that small changes can produce large effects at a so-called ‘tipping’ point [21, 31]. Furthermore, the loop in the bottom of Fig. 1 visualises the hypothesis that realized changes may shift system norms toward a focus on health and well-being, thereby creating momentum for additional HP changes [30]. Finally, a moderating effect of the context on child outcomes is visualised in the right-top of the figure: even when schools implement similar changes, the impact may differ by school [13].

### Participating schools (S1-S4)

The four schools were members of the regional educational board ‘Movare’ situated in the Parkstad region in the southern part of the Netherlands. This region has a low average socio-economic status (SES), and unhealthy behaviours and overweight are highly prevalent compared to the rest of the Netherlands [32, 33]. More information on the recruitment of the schools and participants is described in Willeboordse et al. [19].

### The Healthy Primary School of the Future

The worrying increase in unhealthy behaviours among their schoolchildren and the fragmentation of school health promotion, induced Movare to initiate collaboration with the regional Public Health Services and Maastricht University. Together they developed the idea for the HPSF initiative [19]. The provincial authorities supported the initiative financially. The two changes (providing a lunch each day and structured daily PA session) were implemented by external pedagogical employees (PE) provided by childcare organisations, to avoid increasing the workload of teachers. This integration of the childcare organisation during school hours, is intended to change the school’s organisation in a sustainable way. The aim for the future is to bring school and childcare closer together and thereby create an integrated day for children, whereby children are supervised by the same people prior, during and after school hours. The above mentioned commitment of schools and child care organisations to continued implementation, also includes this employment of external pedagogical employees during school hours.

The lunch products were provided by catering services and instructions for PA sessions were provided by a sports and leisure organisation. The schools involved teachers and parents in the adoption decision and the process of adapting the two changes to the school context. The schools decided to start implementation of HPSF only if they had full teacher support, which was orally assessed during team meetings, and at least 80% parental support, which was assessed by a paper-based survey, asking whether they support the change, and if not, why. Each school selected a teacher as school coordinator, who managed HPSF in their school. A PE coordinator per school acted as contact person for all external PE in that school. A health promoter from the regional Public Health Services was assigned to each school to provide support when needed. In this pilot, researchers from Maastricht University monitored and fed back results to the schools to support the processes of change. Each school initiated regular meetings to discuss their processes of change, such as meetings between the school coordinator and PE coordinator, and working groups with teachers and parents, as well as children’s voice groups. The health promoters of the four schools also met regularly to keep each other updated on the on-going processes of each school. Overarching the four schools, the HPSF initiative was led by a project leader from Movare and an executive board with representatives of the three collaborating organisations, including the project leader. A project team was created with representatives of all partners involved: the four schools, Movare, regional Public Health Services, Maastricht University, the Limburg provincial authorities, childcare organisations, the caterer, and the sports and leisure organisation. More details about the HPSF initiative were published elsewhere [19].

### Mixed methods

#### Interviews

Qualitative in-depth data were collected using semi-structured interviews. At the end of the first two academic years (2014/15 and 2015/16), interviews were held in each school with the school coordinator and the school health promoter together. The interviews aimed to get an overview of the school’s current HP elements (school routine, policy, education, environment), and an understanding of any dominating organisational issues. Notes were taken during these interviews, and each interview was summarized afterwards. The summaries were checked by the interviewees, and fed back to the project team. At the end of the second year of implementation, interviews were held separately with each school coordinator (*n* = 4), PE coordinator (n = 4), school health promoter (n = 4), and the project leader (*n* = 1). Topics explored included the HPSF process of development and implementation, factors influencing this process (coordination, team cohesion, bottom-up involvement, external support, and momentum-effect), adaptations in the school context as a response to HPSF, and the extent to which HPSF was integrated in the school after two years. These interviews were audiotaped, transcribed verbatim, and member-checked.

#### Observations

A researcher participated, observed, and took notes in the four schools and during all meetings of the project team and meetings of the health promoters. The aim was to learn about school dynamics, and to see and hear factors influencing the implementation process (rather than as a form of fidelity assessment). To create an open view, no observational checklist was used by the researcher. During school visits, the researcher randomly talked to school staff and children to hear about their experiences and perceptions regarding HPSF. Observations took at least one full week each year during effect measurements and regular visits (at least once every three months) to each school during the year. Notes were taken during and immediately after visiting the school.

#### Barrier questionnaire

The presence of perceived potential barriers for HPSF were collected by a 46-item questionnaire, distributed by e-mail, that all teachers and external PE were asked to complete digitally or by writing. The questionnaire was based on the Measurement Instrument for Determinants of Innovations (MIDI), a Dutch validated questionnaire developed by Fleuren et al. [25]. Items are formulated as a statement regarding barriers for HPSF related to the innovation, implementers, organisation, or socio-political context. Responses to each statement ranged from 1 (totally disagree) to 10 (totally agree). Statements with an average score below 6 were defined as potential barriers. This corresponded to the grading system used in Dutch primary schools, which also uses a range from 1 to 10 for school tests, and scores below 6 as insufficient or fail. The questionnaire was completed once during the development year, and twice a year during the two years of implementation. To obtain data about dominating organisational issues, and factors influencing the process of development and implementation of HPSF, the questionnaire included open questions, e.g. ‘*Which five factors are in your opinion important to make HPSF successful?*’

#### Practices questionnaire

A questionnaire, based on and used in previous work by Gevers et al. [34] and O’Connor et al. [35], was used annually at the beginning of the academic year to assess nutrition- and PA-related HP practices of teachers and parents, such as modelling behaviour, and encouragement. All teachers received the questionnaire whereas parents only received it when they had signed the consent form (68%). The paper-based teacher questionnaire consisted of 30 items; the digital parent questionnaire consisted of 23 items. Each item described a practice by using a statement, followed by some examples. Participants responded on a Likert scale from 1 (completely disagree) to 5 (completely agree).

#### Minutes of meetings

Minutes were collected of the meetings of the project team, the health promoters, the working groups with parents and teachers, and the children’s voice groups. Data derived from these minutes provided qualitative, in-depth information about the development and implementation of HPSF in each school and any experienced influencing factors.

### Analyses

Thematic analyses were conducted of the qualitative data from the interviews, observations, and minutes [36]. Data were coded into themes based on the programme theory using NVivo (version 11.0). During this coding process, themes were reviewed several times to see if they still worked in relation to the data. After all data were coded, subcategories were created per theme if necessary, and when possible a distinction between inhibiting and promoting was made for the influencing factors. Next, the coded text was retrieved to create an overview per theme (or per subcategory) with the findings split up into the four schools to study similarities and differences. Furthermore, for each school, the frequency of similar answers to the open questions of the barrier questionnaire was calculated. Quantitative questionnaire data were analysed using SPSS (version 23). For each time of measurement and separately for each actor, descriptives were calculated per practice (teachers, parents) or potential barrier (teachers, external PE). Standardized effect sizes (Cohen’s d) per school, defined as (mean at follow-up time of measurement minus mean at baseline) divided by standard deviation at baseline, were calculated for the practices. This effect size calculation was presented on top of the pre- and post-mean (SD) per school, to give an indication of the extent of the changes over time and be able to compare them between the schools. Only the teachers/parents who filled in both the questionnaire at baseline and at T1/T2 were included in this calculation. The effect sizes were categorized in accordance with Lipsey’s guidelines [37]: small (0–0.32), medium (0.33–0.55), and large effect (> 0.56).

## Results

### The pre-existing context of the four schools

School days lasted from 8.30 am to approximately 3.00 pm on Monday to Friday, except for Wednesday, when schools finished around 12.30 pm (Table 2). The schools had a 15-min morning break when children went outside for free play and ate their own brought morning snack. Lunch break time varied between 30 and 60 min across schools: 15 min’ lunch, when they could eat their own brought sandwiches, and 15–45 min of free play outside after lunch. These routines were comparable to other primary schools in the Netherlands. Physical education classes consisted of approximately 60 min/week, except for School 3 (S3), which had 120 min/week. All schools had a sports hall on-site or within walking distance and had several PA possibilities in the schoolyard and the neighbourhood. All schools, except S3, had limited HP policy and education.

**Table 2:** HP elements in the four schools

	School 1	School 2	School 3	School 4
School routine	Prior to HPSF: •Lunch break time: 45 min •Children bring their own packed lunch. Implemented in Y1: vProvided healthy lunch and mid-morning snack •Structured PA and cultural sessions during lunch break •Increased lunch break time to 105 min.	Prior to HPSF: •Lunch break time: 30 min •Children bring their own packed lunch. Implemented in Y1: vProvided healthy lunch and mid-morning snack •Structured PA and cultural sessions during lunch break vIncreased lunch break time to 95 min.	Prior to HPSF: •Lunch break time 45 min •Children bring their own packed lunch. Implemented in Y1: •Structured PA and cultural sessions during lunch break.	Prior to HPSF: •Lunch break time: 1 h •Children bring their own packed lunch or go home for lunch. Implemented in Y1: •Structured PA and cultural sessions during lunch break
Policy	Prior to HPSF: vLimited to no HP policy. Implemented in Y1: •Birthday treat policy vWater policy	Prior to HPSF: •Limited to no HP policy. Implemented in Y1: •Water policy Implemented in Y2: vBirthday treat policy	Prior to HPSF: •Birthday treat policy •Water policy	Prior to HPSF: vLimited to no HP policy.
Education	Prior to HPSF: •Limited to no HP education. •PE classes once a week. Implemented in Y2: •Educational lunch	Prior to HPSF: •Limited to no HP education. •PE classes once a week. Implemented in Y1: •Educational lunch Development phase: •Educational programme on healthy lifestyle.	Prior to HPSF: vHealthy lifestyle education programmes. •PE classes twice a week.	Prior to HPSF: •Limited to no HP education. •PE classes once a week.
Environ-ment	Prior to HPSF: •Once a week fruit from local supermarket. Implemented in Y1: •Providing water bottles. Implemented in Y2: •Vegetable garden in neighbourhood.	Prior to HPSF: - Implemented in Y1: •Providing water bottles. Developmental phase: vVegetable garden	Prior to HPSF: •Active Living: PA-friendly schoolyard. •JOGG: providing water bottles. •RiskCare: offered health-promoting programmes for parents and children and supported the healthy lifestyle education programme. Implemented in Y2: •Vegetables in the schoolyard.	Prior to HPSF: •EU-school-fruit: Offered fruit twice a week.

Abbreviations: HP = Health Promoting; HPSF = Healthy Primary School of the Future; PA = Physical Activity; PE = Physical Education; Y1 = Year 1 of implementation (academic year 2015/16); Y2 = Year 2 of implementation (2016/17)

The teacher practices questionnaire prior to HPSF was completed by 96% of all teachers (S1: 100%; S2: 100%; S3: 75%; S4: 100%). Some of most prevalent nutrition-related practices of teachers prior to HPSF were encouraging the children to eat healthy foods (mean scores between 4.3–4.7), which was especially high in School 1 (S1), S3, and School 4 (S4) (Additional file 1). In S1 and School 2 (S2) the nutrition-related practice of teachers that was also much prevalent was adhering to school’s nutrition-related policy, whereby both schools had a mean score of 4.5. Moreover, also most prevalent in S2 was having clear healthy routines/habits (mean score (SD): 4.3 (0.98)), in S3 educating children on nutrition (mean score (SD): 4.6 (0.70)) and in S4 involving children in healthy nutrition (mean score (SD): 4.3 (1.03)). Some of most prevalent PA-related practices of teachers was creating sufficient access to PA (mean scores between 4.2–4.6), which was especially high in S2, S3 and S4 (Additional file 1). In S3 and S4 the PA-related practice of teachers that was also much prevalent was educating children about PA (mean scores (SD) in S3: 4.7 (0.68); and in S4: 4.5 (0.80)). Moreover, also most prevalent in S1 was having PA-friendly equipment available (mean score (SD): 4.4 (1.03)) and in S2 encouraging children to become physically active (mean score (SD): 4.5 (0.64)). The parental practices questionnaire prior to HPSF was completed by 66% of all the parents who had filled in the consent form (S1: 76%; S2: 56%; S3: 60%; S4: 67%). Most prevalent HP practices of parents at home were similar in all schools: making healthy foods available (mean score between 4.3–4.5) and encouraging their child to eat healthy foods (mean score between 4.3–4.5), having PA-friendly equipment available (mean score between 4.2–4.4), and encouraging their child to become physically active (mean score between 4.2–4.3) (Additional file 1).

Data from the barrier questionnaire revealed that main potential barriers prior to HPSF, generally perceived by external PE, were a lack of time required for implementation (teachers (T): mean score between 4.9–6.9; PE: mean score between 4.6–8.4), limited training opportunities (T: mean score between 6.8–7.9; PE: mean score between 4.5–7.7), and limited available personnel (T: mean score between 6.4–7.5; PE: mean score between 4.0–7.1) (Additional file 2).

In addition, each school had their own specific situation. S1 (26 teachers, 324 children) was a merger of two separate schools, both of which were faced with declining numbers of children. The two schools moved to a new building at the start of HPSF (Nov. 2015). Even though the merger created more work and a distracted focus, it also provided a natural opportunity to make a new start. S2 was also undergoing a merger process, planned for September 2016. For this merger the school building had to be renovated, so they had to move to a temporary location with limited PA possibilities in and around the school from November 2015 to September 2016. Before the merger, the school consisted of 15 teachers and 234 children; after the merger in September 2016, there were 23 teachers and 347 children. S3 (16 teachers, 233 children) had to deal with a major staff turnover at the start of HPSF. It had been participating in several other projects: 1) the Active Living project (prior to HPSF), in which they had changed their schoolyard to improve PA possibilities [31], 2) the JOGG (Youth on Healthy Weight) initiative, in which they had changed their school’s water policy and provided free water bottles for all children, and 3) a project of RiskCare, a local private obesity prevention organisation, in which they received support for training teachers to educate healthy lifestyle lessons. S4 (21 teachers, 389 children) joined HPSF later than the other three schools, i.e., at the end of academic year 2014/15. The school had been participating in the project of EU fruit, in which the school received fruit for all children twice a week.

### Process of change

#### Development and implementation of HPSF

#### Two top-down HP changes

Parental support for HPSF in S1 (89%) and S2 (88%) was high; they also had 100% teacher support. S3 had no unanimous teacher support and 68% parental support, mainly due to criticisms of the lunch. A fourth school dropped out because of a lack of bottom-up support. Due to these differences in support, HPSF was split up into two versions: 1) implementation of the provided lunch and the structured PA sessions, and 2) implementation of the structured PA sessions only. S1 and S2 continued with the first version of HPSF. Since the main criticism in S3 was on the lunch, this school decided to continue with only a focus on PA. After the withdrawal of the fourth school, another school from the same educational board was recruited. For this reason, this ‘new’ S4 did not go through a decision process with teachers and parents due to limited time as they joined the initiative at the end of the school year. They also focused only PA. In S1 and S2, the time for having lunch was increased to 20–30 min (Table 2). The caterer developed a lunch menu cycle that changed every ten weeks, in which at least 80% of the products met the advice of the Dutch Health Council [35]. A mid-morning snack, consisting of fruits and/or nuts, was also provided. The lunch, a bread-based cold meal, was typically Dutch. The PA sessions were carried out in the schoolyard and, when available and needed, in parks, forest, and/or sports hall in the neighbourhood. All schools collaborated with sport clubs or other external partners to offer specific activities. The external PE of S1 and S2 were assigned to the same class for the whole year; the external PE of S3 and S4 were assigned to an activity. A sports and leisure organisation supported the external PE during implementation when needed, and after a year they provided a training course (8 sessions of 2 h) to supply them with additional tools on how to motivate children for active participation during the PA sessions.

#### Additional HP changes

Schools were informed about possible additional HP changes using a ‘fruit basket’ model designed by the researchers, which consists of a continuously expanding overview of available evidence-based structural HP changes [23]. Water bottles were provided to the children in S1, S2 and S4 (Table 2). S1 and S2 created a school water policy. S4 gave the bottles to the children to take home and did not change their policy. S1 and S2 changed their school’s policy on birthday treats. S2 implemented a once-a-week educational lunch. Due to limited structure, the health promoters developed short lessons for this educational lunch based on evidence-based educational healthy lifestyle programmes, which improved the content and structure. As a result of this, S1 also started to use the lessons. The school coordinators of S1, S2, and S3 decided to investigate possibilities for a vegetable garden in their schoolyard or neighbourhood. The S2 school coordinator aimed to start a HP educational programme in the next academic year (2017/18).

#### Influencing factors: interactions between HPSF and the school context

#### Coordination

According to the school coordinators, the main promoting factor to coordinate HPSF properly was support from and good collaboration and communication with the PE coordinator in the school (Table 3). The school coordinators also felt that having sufficient time during lunch breaks greatly improved the implementation process due to increased focus on their coordinating tasks. The S1 and S4 school coordinators noted that they were mainly busy with the daily practical issues around the lunch and structured PA sessions, which created a limited focus on the overall coordination of HPSF. This limited overall focus was perceived by the health promoters as inhibiting for the initiation of additional HP changes.

**Table 3:** Influencing factors on HPSF in the four schools

	School 1	School 2	School 3	School 4
Coordination	−/+ Mainly focus on lunch break changes, modest collaboration between school coordinator and PE coordinator	++ Overall focus, optimal collaboration between school coordinator and PE coordinator	- Limited time and focus, limited collaboration between school coordinator and PE coordinator	+ Mainly focus on lunch break changes, optimal collaboration between school coordinator and PE coordinator
Team cohesion	+ External PE assigned to class, contact and collaboration improved over time, also due to annual party for whole team	++ External PE assigned to class contact and collaboration improved over time, also due to training course for whole team and much focus and efforts from coordinators	- More classes than external PE, external PE divided by activity, limited contact between external PE and teachers, meeting helped to get to know each other.	−/+ More classes than external PE, external PE divided by activity, contact between external PE and teachers when needed, meeting helped to get to know each other.
Bottom-up involvement: development	++ Full year for development, teachers and parents involved, unanimous teacher support, 89% parent support	++ Full year for development, teachers and parents involved, unanimous teacher support, 88% parent support	−/+ Full year for development, teachers and parents involved, no full teacher support, 68% parent support	- Two months for development, teachers and parents not fully involved in development process
Bottom-up involvement: implementation	−/+ Children voice group, parental volunteers, some additional changes with involvement of parents and teachers, teachers’ assumption that HPSF consists only of the lunch break changes and does not involve teacher participation	−/+ Children voice group, parental volunteers, some additional changes with involvement of parents and teachers, teachers’ assumption that HPSF consists only of the lunch break changes and does not involve teacher participation	- Children voice group, no parental volunteers, teachers’ and parents’ involvement limited, teachers’ assumption that HPSF consists only of the lunch break changes and does not involve teacher participation	- Children voice group, parental volunteers, teachers’ assumption that HPSF consists only of the lunch break changes and does not involve teacher participation
External support	++ Many different external partners involved and supporting the schools in all aspects of HPSF.	++ Many different external partners involved and supporting the schools in all aspects of HPSF.	++ Many different external partners involved and supporting the schools in all aspects of HPSF.	++ Many different external partners involved and supporting the schools in all aspects of HPSF.

Abbreviations: *HPSF*= Healthy Primary School of the Future, *PE*= Pedagogical employees

#### Team cohesion

According to teachers and external PE, important factors for success were the availability of external PE and the collaboration between PE and teachers (Additional file 3). However, particularly in the first year of implementation, the relationship and communication between external PE and teachers was suboptimal: they had to get used to each other, and their mutual responsibilities were not completely clear. Limited time available for formal meetings and limited permanent external PE impeded feedback opportunities and inhibited the process of creating good collaboration and communication. To improve this, all schools created one or more occasions for (in)formal contact to get to know each other and to create one team. Two schools (S1 and S2) had to deal with a merger during HPSF, which also increased the need for team cohesion. These schools had put extra efforts to create occasions for contact, by organising a party for everybody (S1) or introducing a training relevant for all teachers and PE (S2). Finally, PE coordinators indicated that external PE being assigned to a class promoted the cooperation with teachers and the relationship with children, while being assigned to an activity inhibited it.

#### Bottom-up involvement

To create sufficient support for implementation, it was perceived as important by the school coordinators to involve all actors immediately at the start of the decision and development process, especially parents who were critical of the HPSF approach. To build this involvement, the schools had started with an enthusiastic team of teachers: their positive attitude towards HPSF in formal and informal conversations with parents stimulated parents’ enthusiasm, which created a positive atmosphere around HPSF and improved involvement. Teachers and external PE perceived bottom-up involvement and everyone’s enthusiasm throughout the years as one of the main factors to make the two changes successful. However, the school coordinators also perceived that the involvement of teachers and parents to further improve school health promotion by additional HP changes faded after the two changes were successfully integrated in the school and had become part of the daily functioning of the school system. This was also seen in the responses to the barrier questionnaire: the majority of teachers reported that they could not fill out most of the statements, as they did not feel that it applied to them because they were not involved in implementing the two changes, which they considered the only components of HPSF. This result was also fed back to and discussed in the project team. According to the health promoters and project leader, this lack of perceived involvement was a key inhibiting factor to the implementation of additional HP changes.

#### External support

Support from external partners was highly appreciated and all schools indicated it as essential for the success of HPSF. Both the availability of external PE provided by childcare organisations and the practical support provided by a sports and leisure organisation, the caterer, and the health promoters were considered being essential. Perceived promoting aspects for collaboration with external partners were regular feedback between the practical level of each school (the implementers) and the project team, direct communication with each other, and clear responsibilities of each person. The researcher’s support was perceived as valuable when the provided feedback was to the point and tailored to each specific school. The coordinators perceived that the fruit basket model helped the schools to think of additional HP changes that are structural and evidence-based.

#### Momentum

Participants reported that implementation of the lunch in S1 and S2 was key in creating momentum to implement additional HP changes. The school coordinators of these schools indicated that the lunch made it easier to implement the water bottles because children did not have to bring any food or drinks to school anymore, and it created a good opportunity to change school policy around birthday treats. The health promoters indicated that the lunch made it also easier (compared to other schools in the region) to implement additional HP changes due to an improved health-promoting mind-set. This momentum effect was not observed in the four schools for the PA sessions.

### Integration of HPSF in the school context and perceived impact

During the two years of implementation, a decline was observed in the number of perceived potential barriers among both external PE and teachers in S1, S2, and S4. This seems to indicate more integration of HPSF into the schools. An opposite result was found in S3, where the number of perceived barriers indicated by external PE increased during the implementation period. Factors in S3 that continued to be perceived as barrier throughout almost all measurements were: perceived outcome importance (mean score of the different measurements between 3.0–5.6), observability (mean scores between 3.5–5.9), adaptability (mean scores between 3.7–7.0), availability of materials (mean scores between 3.6–6.8), and support of parents (mean scores between 3.0–3.7).

Looking at the perceived impact of HPSF, some similarities were found across the schools, mainly regarding perceptions of improved health behaviours of children and improved healthy practices of teachers. All schools described perceptions that since HPSF, the children created and managed their own activities more easily during free play, they were less bored during recess time, and fewer conflicts happened, which contributed to a calmer environment. Fewer impacts were mentioned regarding the school’s way of working to create change in the whole school system, as the main focus was on the two changes. Furthermore, interviewees from S1 and S2 reported that lunchtime had become a more socializing moment, children ate a wider variety of foods and became more open to trying unfamiliar products. Issues regarding children’s dietary behaviours became clearer and were easier to discuss with parents.

Overall, teachers’ practices changed in a more favourable direction (Additional file 1). Large effect sizes were found for nutrition-related practices in S1 and S2, e.g., discussing (S1: effect size (ES) = 0.07; S2: ES = 0.81) and educating about nutrition (S1: ES = 0.38, S2: ES = 0.91), and monitoring children’s dietary behaviours (S1: ES = 1.16, S2: ES = 0.09). In S1 and S4 large effect sizes were found for teachers’ PA-related practices, such as involving children in PA (S1: ES = 0.85, S4: ES = 0.59), and having routines/habits for PA (S1: ES = 0.86, S4: ES = 0.62). Teacher’s modelling behaviour regarding nutrition and PA changed in S1 (nutrition: ES = 0.35, PA: ES = 0.69), S2 (nutrition: ES = 0.47, PA: ES = 0.36), and S4 (nutrition: ES = -0.11, PA: ES = 0.33) mostly in a favourable direction, with often medium effect sizes. Effect sizes in S3 could not be determined due to a limited sample size as only four teachers filled out the questionnaire at both baseline and follow-up. Some parental practices changed, though none with a large effect size (Additional file 1). Medium effect sizes for parental practices were found for educating about PA (ES between − 0.05 – 0.36) and emotional feeding (ES between − 0.33 – 0.14), which mostly changed in a favourable direction; involving children in PA (ES between − 0.33 – 0.04) and making PA-stuff available (ES between − 0.48 – 0.00) changed mostly in an unfavourable direction.

In addition, school-specific perceived impacts for S1 include: the school team became closer, and a school day was seen more as an entirety that fits together. It was also perceived that the children became more creative, worked more together, and talked differently about healthy nutrition in school: it became a part of their identity and not just some school activity. In S2, the school coordinator indicated that teachers’ focus on healthy behaviours had improved, e.g., teachers used more often healthy lifestyle topics in their lessons, they tended to keep each other updated regarding healthy lifestyle news items, and they were more aware of their own modelling behaviour. It was perceived in S3 that children became more enthusiastic about PA and going outside; teachers used more often healthy nutrition topics in their lessons. The interviewees of S4 indicated that teachers were more aware of possibilities for PA in school, and their interest in how to improve children’s dietary behaviours had increased slightly.

## Discussion

The current study explored the implementation of HPSF and the processes through which HPSF and the school context adapt to one another over time. Even though similarities existed since the schools are all part of the Dutch school system, the schools dealt with different contextual issues. These differences in context also influenced the evolution, implementation and impact of HPSF, demonstrating the importance of a contextual approach [10, 15].

Top-down advice and external practical support was perceived as helping the schools to initiate a positively disruptive change. Bottom-up involvement was needed throughout the process to contextualize and optimize changes and to create ownership. Sufficient coordination and communication at the school level, the availability of external PE, team cohesion, and feedback loops among all actors involved enhanced the implementation of the changes. These findings of the current study, in which we used a systems approach, are consistent with and add to the findings and recommendations of previous studies which also point to the importance of feedback, external support, clear coordination and communication, and bottom-up involvement for sufficient adoption and implementation of school health promotion programmes [9, 28, 29, 38]. The current study further extends the knowledge by, among other things, insight on creating disruption in the schools. Modifying the school lunch acted as an entry point for health improvement action due to the particular Dutch context in which provision of lunch by schools is not typical practice [30], and appeared to act as a catalyst for additional HP changes. Most of the implemented additional HP changes were described as being facilitated by the provided lunch.

The PA sessions did not have this disruptive effect in the schools, also not in S1 and S2. Two explanations can be given for this. First, while the lunch acted as a highly visible change in practice with everyday implications for parents and teachers, the PA sessions did not appear to have such a visible impact on parents and teachers, it’s perceived influence being primarily with the children themselves. Perhaps due to the more limited number of stakeholders impacted by this change, the PA sessions did also not lead to much discussion among the people involved. Second, the topic of the change could also be a reason. It was observed in the different data sources that changes related to nutrition seemed to come much closer to essential aspects of parenting than changes related to PA. Altogether, this seems to indicate that both the topic of the change and the impacts of the disruption across multiple stakeholder groups is important.

Adaptations in the school context also occurred in teachers’ HP practices: in S1 and S2, teachers’ practices changed after two years of HPSF, several with a large effect size. Interestingly, looking at the mean, SD and effect sizes of the modelling practices of teachers, only moderate improvements can be seen, even though during the interviews the schools indicated a specific focus on modelling [39]. However, since no statistical tests were conducted, no hard conclusions could be drawn and further analysing is needed. Furthermore, the findings showed that aspects of the health promoting school concept [7], such as creating a HP environment or participation of parents and children, were in the first two years of implementation often directly related to the two top-down changes. This means that even though several impacts on health behaviours were perceived, there is still room for improvement to further increase the impact on the whole school system. However, as also indicated in the programme theory, this system change takes time due to the feedback loops that need to develop in the system.

The main recommendations resulting from this study were related back to the programme theory and combined into five key learning points for research and practice (Table 4). Four learning points can hereby be seen as conditions that were successful in the participating schools to create a major change that should lead to disruption; the last learning point is related to how to use a created disruption.

**Table 4:** Key learning points

How to create a disruption?		
1.	Creating a disruption in a school takes time and needs bottom-up involvement	This learning point shows the importance of bottom-up involvement as indicated in the programme theory. Moreover, it also relates back to the several loops of feedback arrows between HPSF and the school context. In the four participating schools was seen that creating bottom-up involvement immediately at the start of the developmental phase took time but seemed to increase people’s ownership and support. Implementation of changes also took time as the school needed to find a new way of working in the school to create for example a good collaboration between the teachers and the external PE.
2.	Regular contact among all actors is required to get to know each other and to manage expectations.	This learning point relates back to the importance of sufficient coordination and team cohesion. In the four participating schools was seen that regular contact between the people involved, not only to discuss the content, but also to get to know each other, helped to create more understanding and feelings of mutual support. Regular contact between teachers and external PE improved team cohesion in the school, which enhanced implementation. In particular, communication about expectations of everybody’s responsibilities appeared to be important.
3.	Top-down advice and external practical support are important for creating a disruption.	This learning point shows the importance of external support, as indicated in the programme theory. In the four participating schools was seen that top-down advice and practical support from external partners helped the schools by providing personnel, money, materials, and knowledge.
4.	To contextualize and realize changes feedback loops are required among all involved actors.	This fourth learning point does not only relate back to the several loops of feedback arrows in the programme theory between HPSF and the school context, it also shows the importance of external support and the involvement from bottom-up. In the four participating schools was seen that feedback loops in school among staff, children, and parents made a change better fit into the school context with its specific needs and wishes. Feedback loops between school and external partners made the external support to school, to realize the changes, as efficient as possible.
How to use a disruption?		
5.	A disruption is useful for implementing additional HP changes on the same topic.	This last learning point relates back to the loop in the bottom of the programme theory which indicates the momentum-effect. In this study the provided lunch disrupted the existing dynamics in the school and created momentum for nutrition-related additional HP changes, as people perceived these additional HP changes as something that came along with the provided lunch. The health promoters felt that due to the lunch in S1 and S2, additional nutrition-related HP changes were implemented with less discussion and easier acceptance, compared to other schools in the region, due to an improved health-promoting mind-set. However, the lunch did not create momentum for not nutrition-related initiatives, i.e., PA-related.

### Strengths and limitations

The results should be considered in light of the study’s strengths and limitations. A strength of the study is that due to using mixed methods, we were able to employ the principle of data triangulation and combine the accuracy of quantitative questionnaires with in-depth insights afforded by interviews, observations, minutes, and open questions. Triangulation is a strategy that facilitates validation of data through cross-verification from different sources [40], and is stimulated by other researchers to employ in process evaluations [12]. Using CARA meant that the researchers in this pilot were not external observers, but actively participating partners in the initiative. The researchers not only evaluated the processes of change by using mixed methods, but also supported the schools in their processes. Researchers’ support in this pilot consisted of offering their knowledge and expertise and by providing regular feedback based on the results of the mixed methods. However, schools always decided themselves what to do with this information. The active participation of researchers helped the schools to improve their changes, and it gave the researchers a deep and honest insight into each school’s process of change, as a relationship of trust was built up with the people in the school. However, due to this research approach, the researchers interfere with the implementation processes and are not fully objective anymore, which can be seen as limitation. By conducting the process evaluation prior to the effect evaluation, where a quasi-experimental study design was used, we were able to combine the best of two worlds: the advantages of a researcher involved in the process of change without knowing the effects, and studying the effects objectively by the quasi-experimental design [13].

Another limitation of the study is that it was impossible to fully assess and understand all aspects of each school’s context and process of change due to limitations in time, resources, and participant burden [13]. To deal with this issue, we followed recent research suggestions to mainly focus on the factors that are indicated as relevant for improving school health promotion [8, 10, 24, 25]. Finally, the four pilot-schools could be classified as early adopters, who were open for system change. Scaling up the HPSF initiative should also include schools that are less open for change. Bottom-up involvement from the start is hereby crucial to create ownership and support in the school. Communicating about the benefits experienced by the early adopters could help to increase the engagement in these schools [41]. When scaling-up, the support provided by the researchers should be maintained to contribute to the process of feedback in the schools. This supporting role might be incorporated in the work of the health promoter who is connected to the school.

## Conclusions

Taking the studies’ limitations and strengths into account, it can be concluded that creating an initial, highly visible and well supported positive disruption to improve school health can act as a catalyst for wider school health promotion efforts. Conditions to create a positive disruption are enough time, and sufficient bottom-up involvement, external support, team cohesion and coordination. The focus should be on each specific school, as each school has their own starting point and process of change.

## Additional Files

Additional file 1: Nutrition- and PA-related practices. Description: Nutrition- and PA-related practices of teachers and parents (DOCX 89 kb)

Additional file 2: Perceived potential barriers for HPSF. Description: Presence of potential barriers for HPSF according to teachers and external pedagogical employees (DOCX 83 kb)

Additional file 3: Perceived important factors for success of HPSF. Description: Perception of teachers and external pedagogical employees regarding important factors for success (DOCX 19 kb)

## Abbreviations

CARA: Contextual Action-oriented Research Approach
HP: Health Promoting
HPSF: Healthy Primary School of the Future
PA: Physical Activity
PE: Pedagogical Employees
RQ: Research Question
S: School
SES: Socio-Economic Status
UM: University of Maastricht
