# Supplementary material for: Process evaluation of the healthy primary School of the Future: the key learning points
Source: BMC Public Health. 2019 Jun 6;19:698. doi: 10.1186/s12889-019-6947-2 (PMC6554901; doi:10.1186/s12889-019-6947-2)
Supplement: Supplementary file 1 — Nutrition- and PA-related practices. Description: Nutrition- and PA-related practices of teachers and parents (DOCX 89 kb) [file 12889_2019_6947_MOESM1_ESM.docx]

**Additional file 1. Nutrition- and PA-related practices**

**Additional file 1a. Nutrition- and PA-related practices of teachers**

|  | | | **T0** | | **T1** | | | **T2** | | |
| --- | --- | --- | --- | --- | --- | --- | --- | --- | --- | --- |
|  |  |  | **N** | **Mean (±SD)** | **N** | **Mean (±SD)** | **Effect size*** | **N** | **Mean (±SD)** | **Effect size*** |
| **Nutrition-related practices** | | | | | | | | | | |
| **Healthy modelling**  **(1-5)** | I consciously eat healthy food products when the children are around.  *For example, by eating fruit when the children are around.* | **S1** | 26 | 4.4 (±0.80) | 24 | 4.3 (±0.99) | -0.17 | 29 | 4.5 (±0.74) | 0.35 |
|  |  | **S2** | 15 | 4.3 (±1.10) | 23 | 4.8 (±0.39) | 0.59 | 19 | 4.7 (±.56) | 0.47 |
|  |  | **S3** | 9 | 4.2 (±0.97) | 15 | 4.4 (±1.12) | - | 8 | 4.1 (±0.99) | - |
|  |  | **S4** | 22 | 4.1 (±1.05) | 25 | 4.2 (±1.07) | 0.23 | 24 | 4.0 (±0.89) | -0.11 |
| **Visibility**  **(1-5)** | I ensure that healthy food products are visible for the children (e.g. pictures).  *For example, by hanging up pictures of fruit in the classroom. Or by hanging up visualisations (e.g. mind map) of group sessions about healthy foods.* | **S1** | 26 | 3.7 (±1.23) | 23 | 3.4 (±1.19) | -0.11 | 29 | 3.7 (±1.31) | 0.27 |
|  |  | **S2** | 15 | 3.6 (±1.24) | 22 | 4.1 (±1.08) | 0.32 | 19 | 4.1 (±0.71) | 0.35 |
|  |  | **S3** | 10 | 4.0 (±0.82) | 15 | 3.4 (±1.35) | - | 8 | 3.0 (±1.07) | - |
|  |  | **S4** | 21 | 3.7 (±1.56) | 25 | 3.7 (±1.54) | 0.00 | 24 | 3.7 (±0.96 | -0.04 |
| **Encour-agement**  **(1-5)** | I encourage the children to eat healthily.  *For example, by encouraging the children to bring and eat vegetables as a midmorning break. Or by encouraging them to first eat their lunch and afterwards their sweet snack.* | **S1** | 26 | 4.5 (±0.51) | 24 | 4.5 (±0.59) | 0.09 | 28 | 4.6 (±0.56) | 0.42 |
|  |  | **S2** | 15 | 4.3 (±0.88) | 23 | 4.9 (±0.55) | 0.75 | 19 | 4.9 (±0.46) | 1.08 |
|  |  | **S3** | 10 | 4.7 (±0.48) | 15 | 4.5 (±0.52) | - | 8 | 4.5 (±0.54) | - |
|  |  | **S4** | 22 | 4.5 (±0.60) | 25 | 4.4 (±0.49) | -0.08 | 24 | 4.3 (±0.75) | -0.17 |
| **Involving**  **(1-5)** | I involve the children in things that concern a healthy diet.  *For example, by involving the children when handing out fruit. By involving children in a vegetable garden. By letting children taste different sorts of fruit during the lesson.* | **S1** | 26 | 4.4 (±0.63) | 24 | 4.0 (±0.46) | -0.58 | 29 | 4.5 (±0.63) | 0.57 |
|  |  | **S2** | 15 | 4.1 (±1.16) | 22 | 4.5 (±0.67) | 0.29 | 19 | 4.5 (±0.51) | 0.31 |
|  |  | **S3** | 10 | 4.3 (±0.95) | 15 | 4.1 (±0.92) | - | 8 | 4.4 (±0.52) | - |
|  |  | **S4** | 22 | 4.3 (±1.03) | 25 | 4.2 (±0.82) | -0.19 | 24 | 4.1 (±0.95) | -0.31 |
| **Discus-sing**  **(1-5)** | I discuss nutrition with the children.  *For example, by discussing the food products they eat and like.* | **S1** | 26 | 4.4 (±0.75) | 24 | 4.3 (±0.44) | -0.25 | 29 | 4.4 (±0.57) | 0.07 |
|  |  | **S2** | 15 | 4.3 (±0.72) | 22 | 4.7 (±0.55) | 0.75 | 19 | 4.7 (±0.45) | 0.81 |
|  |  | **S3** | 10 | 4.5 (±0.53) | 15 | 4.4 (±0.74) | - | 8 | 4.3 (±0.71) | - |
|  |  | **S4** | 22 | 4.2 (±0.61) | 25 | 4.2 (±0.78) | 0.00 | 24 | 4.1 (±0.68) | -0.09 |
| **Educating**  **(1-5)** | I teach the children about nutrition.  *For example, by teaching the children where food comes from. By explaining that eating healthy foods is better than candy or cookies.* | **S1** | 26 | 4.2 (±0.77) | 24 | 4.1 (±0.74) | -0.12 | 29 | 4.5 (±0.69) | 0.38 |
|  |  | **S2** | 15 | 4.2 (±0.68) | 22 | 4.7 (±0.55) | 0.81 | 19 | 4.5 (±0.61) | 0.91 |
|  |  | **S3** | 10 | 4.6 (±0.70) | 15 | 4.2 (±0.86) | - | 8 | 4.1 (±0.64) | - |
|  |  | **S4** | 22 | 4.1 (±0.68) | 25 | 4.4 (±0.82) | 0.51 | 24 | 4.3 (±0.74) | 0.26 |
| **Routines**  **(1-5)** | I ensure that there are healthy habits during moments of eating and drinking in school.  *For example, by taking enough time for lunch and to wait till everyone is finished.* | **S1** | 26 | 4.4 (±0.85) | 24 | 4.5 (±0.59) | 0.16 | 29 | 4.5 (±0.51) | 0.29 |
|  |  | **S2** | 15 | 4.3 (±0.98) | 23 | 4.7 (±0.56) | 0.55 | 19 | 4.7 (±0.48) | 0.76 |
|  |  | **S3** | 9 | 4.2 (±0.83) | 15 | 4.5 (±0.52) | - | 8 | 4.4 (±0.52) | - |
|  |  | **S4** | 21 | 4.2 (±0.81) | 25 | 4.1 (±0.83) | -0.12 | 24 | 4.2 (±0.82) | 0.00 |
| **Monitoring**  **(1-5)** | I try to watch what the children are eating during the day.  *For example, by paying extra attention when a child often eats unhealthy foods as midmorning break or lunch.* | **S1** | 26 | 3.5 (±1.07) | 24 | 4.2 (±0.96) | 0.82 | 29 | 4.5 (±1.30) | 1.16 |
|  |  | **S2** | 15 | 3.5 (±1.13) | 22 | 4.2 (±0.91) | 0.68 | 19 | 3.8 (±1.03) | 0.09 |
|  |  | **S3** | 10 | 3.5 (±1.43) | 15 | 3.6 (±0.83) | - | 8 | 3.8 (±0.46) | - |
|  |  | **S4** | 22 | 2.9 (±1.41) | 25 | 2.9 (±1.71) | 0.00 | 24 | 3.4 (±1.59) | 0.36 |
| **Pressure to eat**  **(1-5)** | I insist that children finish their meal.  *For example, by insisting that children completely finish their lunch.* | **S1** | 26 | 3.3 (±1.09) | 24 | 3.3 (±0.87) | 0.14 | 29 | 3.6 (±1.15) | 0.44 |
|  |  | **S2** | 15 | 3.6 (±0.91) | 22 | 3.5 (±0.96) | 0.28 | 19 | 3.9 (±1.73) | 1.00 |
|  |  | **S3** | 10 | 3.5 (±1.35) | 15 | 3.3 (±0.90) | - | 8 | 3.0 (±0.76) | - |
|  |  | **S4** | 22 | 3.6 (±1.37) | 25 | 3.3 (±1.35) | -0.15 | 24 | 3.2 (±1.47) | -0.36 |
| **Providing feedback**  **(1-5)** | I give feedback to the children concerning their dietary behaviour.  *For example, by commenting when a child often does not finish his/her lunch, often eats unhealthy foods or does not take the time to eat.* | **S1** | 26 | 4.0 (±0.66) | 24 | 4.1 (±0.72) | 0.54 | 29 | 4.2 (±0.69) | 0.86 |
|  |  | **S2** | 15 | 3.9 (±0.80) | 23 | 4.3 (±0.71) | 0.09 | 19 | 4.3 (±0.56) | 0.20 |
|  |  | **S3** | 10 | 3.7 (±1.06) | 15 | 4.2 (±0.56) | - | 8 | 4.1 (±0.35) | - |
|  |  | **S4** | 22 | 4.1 (±0.99) | 25 | 3.6 (±1.22) | -0.58 | 24 | 3.8 (±1.02) | -0.31 |
| **Rules**  **(1-5)** | I strictly follow school policy on nutrition in school and in class.  *For example, by following strictly the policy for celebration treats or the prohibition on energy drinks.* | **S1** | 26 | 4.5 (±0.91) | 24 | 4.7 (±0.64) | 0.26 | 29 | 4.9 (±0.65) | 0.85 |
|  |  | **S2** | 15 | 4.5 (±0.52) | 23 | 4.7 (±0.49) | 0.16 | 19 | 4.7 (±0.58) | 0.35 |
|  |  | **S3** | 10 | 4.4 (±0.70) | 15 | 4.5 (±0.74) | - | 8 | 4.3 (±0.89) | - |
|  |  | **S4** | 22 | 4.2 (±0.96) | 25 | 4.2 (±1.16) | 0.05 | 23 | 4.4 (±0.89) | 0.29 |
| **Unhealthy modelling**  **(1-5)** | I consciously do not eat unhealthy food products when the children are around.  *For example, by eating unhealthy food products in the teachers’ room or at home.* | **S1** | 26 | 4.0 (±1.20) | 24 | 4.3 (±0.82) | 0.23 | 29 | 4.6 (±0.69) | 0.51 |
|  |  | **S2** | 15 | 4.2 (±0.94) | 23 | 4.9 (±0.51) | 0.73 | 19 | 4.9 (±0.62) | 0.45 |
|  |  | **S3** | 10 | 4.1 (±1.10) | 15 | 4.3 (±0.98) | - | 8 | 3.5 (±1.20) | - |
|  |  | **S4** | 22 | 3.5 (±1.18) | 25 | 3.6 (±1.16) | 0.12 | 24 | 3.7 (±1.17) | 0.00 |
| **Instrumental feeding**  **(1-5)** | I reward the children sometimes with unhealthy food products when they did something well.  *For example, by rewarding the children with candy in a certain lesson when they did something really well.* | **S1** | 26 | 2.5 (±1.75) | 24 | 2.0 (±1.08) | -0.21 | 29 | 2.4 (±1.78) | 0.20 |
|  |  | **S2** | 15 | 2.8 (±1.52) | 23 | 2.5 (±1.90) | -0.41 | 19 | 1.5 (±1.17) | -0.78 |
|  |  | **S3** | 10 | 2.8 (±1.48) | 15 | 1.8 (±1.01) | - | 8 | 2.1 (±1.25) | - |
|  |  | **S4** | 22 | 2.9 (±1.39) | 25 | 3.2 (±1.63) | 0.27 | 24 | 2.8 (±1.63) | -0.15 |
| **PA-related practices** | | | | | | | | | | |
| **Avail-ability**  **(1-5)** | I ensure there is PA-friendly equipment available for the children.  *For example, by preparing outdoor toys like a skipping rope or football.* | **S1** | 26 | 4.4 (±1.03) | 24 | 4.2 (±0.83) | -0.05 | 29 | 4.3 (±1.00) | 0.15 |
|  |  | **S2** | 15 | 4.1 (±0.64) | 23 | 4.4 (±0.73) | 0.13 | 19 | 4.3 (±0.45) | 0.14 |
|  |  | **S3** | 10 | 4.3 (±0.48) | 15 | 4.1 (±0.70) | - | 8 | 4.3 (±0.89) | - |
|  |  | **S4** | 22 | 4.1 (±1.07) | 25 | 4.4 (±1.04) | 0.35 | 24 | 4.0 (±1.00) | 0.09 |
| **Visibility**  **(1-5)** | I ensure physical activity is visible in the classroom.  *For example, by hanging up pictures of different kinds of sports in the classroom. By hanging up visualisations (e.g. mind map) of group discussions regarding physical activity.* | **S1** | 26 | 3.6 (±1.50) | 24 | 3.3 (±1.33) | -0.24 | 29 | 4.0 (±1.59) | 0.40 |
|  |  | **S2** | 15 | 3.1 (±1.30) | 21 | 3.7 (±1.55) | 0.30 | 19 | 3.5 (±0.90) | 0.12 |
|  |  | **S3** | 10 | 3.3 (±1.34) | 15 | 3.2 (±1.66) | - | 8 | 4.0 (±1.69) | - |
|  |  | **S4** | 22 | 3.6 (±1.71) | 25 | 3.3 (±1.57) | -0.11 | 24 | 3.1 (±1.32) | -0.25 |
| **Accessibility**  **(1-5)** | I enable the children to be physically active.  *For example, by regularly using energizers in the classroom. By always giving children the opportunity to be physically active even when it is raining outside.* | **S1** | 26 | 4.2 (±0.71) | 24 | 4.1 (±0.45) | 0.07 | 29 | 4.4 (±0.62) | 0.37 |
|  |  | **S2** | 15 | 4.6 (±0.63) | 23 | 4.3 (±0.54) | -0.37 | 19 | 4.6 (±0.51) | 0.26 |
|  |  | **S3** | 10 | 4.6 (±0.52) | 15 | 4.4 (±0.51) | - | 8 | 4.6 (±0.52) | - |
|  |  | **S4** | 22 | 4.5 (±0.67) | 25 | 4.4 (±0.87) | -0.07 | 24 | 4.5 (±0.59) | 0.00 |
| **Encour-agement**  **(1-5)** | I encourage the children to be physically active.  *For example, by discussing the advantages of physical activity. By providing ideas for PA games during breaks and playing these games during PE.* | **S1** | 26 | 4.2 (±0.71) | 24 | 4.1 (±0.58) | -0.08 | 29 | 4.6 (±0.57) | 0.83 |
|  |  | **S2** | 15 | 4.5 (±0.64) | 23 | 4.6 (±0.59) | 0.00 | 19 | 4.4 (±0.61) | -0.13 |
|  |  | **S3** | 10 | 4.5 (±0.71) | 15 | 4.3 (±0.49) | - | 8 | 4.6 (±0.52) | - |
|  |  | **S4** | 22 | 4.2 (±0.81) | 25 | 4.6 (±0.71) | 0.46 | 24 | 4.5 (±0.78) | 0.06 |
| **Rewarding**  **(1-5)** | I reward the children for being physically active.  *For example, by giving the children compliments when they are very physically active during breaks or performed well during PE.* | **S1** | 26 | 4.1 (±0.89) | 24 | 4.0 (±0.55) | -0.05 | 29 | 4.6 (±0.83) | 0.69 |
|  |  | **S2** | 15 | 4.1 (±0.83) | 23 | 4.4 (±0.66) | 0.32 | 19 | 4.1 (±0.71) | 0.24 |
|  |  | **S3** | 10 | 4.1 (±0.99) | 15 | 4.1 (±0.99) | - | 8 | 4.3 (±0.89) | - |
|  |  | **S4** | 22 | 4.1 (±1.11) | 25 | 4.2 (±1.11) | 0.09 | 24 | 4.0 (±1.00) | -0.19 |
| **Involving**  **(1-5)** | I involve the children in things that concern physical activity.  *For example, by giving the children a choice in the kind of energizer for that moment, or the kind of activity during part of the PE.* | **S1** | 26 | 3.8 (±0.91) | 24 | 3.6 (±0.83) | -0.06 | 29 | 4.1 (±0.95) | 0.85 |
|  |  | **S2** | 15 | 4.2 (±0.68) | 22 | 4.1 (±0.64) | 0.00 | 19 | 3.9 (±0.71) | -0.13 |
|  |  | **S3** | 10 | 4.5 (±0.53) | 15 | 4.1 (±0.59) | - | 8 | 4.5 (±0.54) | - |
|  |  | **S4** | 21 | 3.5 (±1.03) | 25 | 4.2 (±0.99) | 0.52 | 24 | 4.2 (±0.78) | 0.59 |
| **Healthy modelling**  **(1-5)** | I am consciously physically active when the children are around.  *For example, by playing outside with the children during breaks, dancing together with the children, or do an energizer together. By commuting to school also in an active way.* | **S1** | 26 | 3.7 (±1.01) | 24 | 3.6 (±0.83) | 0.05 | 29 | 4.0 (±0.98) | 0.69 |
|  |  | **S2** | 15 | 3.9 (±0.92) | 23 | 4.2 (±0.83) | 0.43 | 19 | 3.9 (±0.71) | 0.36 |
|  |  | **S3** | 10 | 3.9 (±0.88) | 15 | 3.7 (±0.96) | - | 8 | 4.1 (±0.84) | - |
|  |  | **S4** | 22 | 3.4 (±1.37) | 25 | 3.7 (±1.31) | 0.14 | 24 | 3.9 (±1.15) | 0.33 |
| **Discus-sing**  **(1-5)** | I discuss physical activity with the children.  *For example, by discussing the different possibilities for physical activity that the children like or dislike. By asking which sports they play in their leisure time.* | **S1** | 26 | 4.1 (±0.74) | 24 | 3.9 (±0.65) | -0.21 | 29 | 4.2 (±0.77) | 0.69 |
|  |  | **S2** | 15 | 4.0 (±0.85) | 23 | 4.4 (±0.50) | 0.44 | 19 | 4.2 (±0.50) | 0.39 |
|  |  | **S3** | 10 | 4.5 (±0.53) | 15 | 4.4 (±0.63) | - | 8 | 4.3 (±0.71) | - |
|  |  | **S4** | 20 | 4.3 (±0.80) | 25 | 4.2 (±0.52) | 0.00 | 24 | 4.3 (±0.76) | 0.15 |
| **Educating**  **(1-5)** | I teach the children about physical activity.  *For example, by teaching the children new options to be physically active or teaching them the rules of games. By teaching the children that physical activity is good for your health.* | **S1** | 26 | 4.2 (±0.63) | 24 | 4.1 (±0.54) | 0.00 | 29 | 4.5 (±0.74) | 1.18 |
|  |  | **S2** | 15 | 4.2 (±0.56) | 22 | 4.2 (±0.61) | 0.00 | 19 | 4.2 (±0.69) | -0.15 |
|  |  | **S3** | 10 | 4.7 (±0.68) | 15 | 4.3 (±0.62) | - | 8 | 4.6 (±0.74) | - |
|  |  | **S4** | 22 | 4.5 (±0.80) | 25 | 4.1 (±0.88) | -0.35 | 24 | 4.3 (±0.74) | -0.19 |
| **Monitoring**  **(1-5)** | I check in general the amount of physical activity of the children during a day.  *For example, by paying extra attention to someone who is not often physically active during breaks or PE.* | **S1** | 26 | 3.5 (±1.11) | 24 | 3.2 (±0.78) | -0.13 | 29 | 3.9 (±1.45) | 0.32 |
|  |  | **S2** | 15 | 3.1 (±1.03) | 22 | 3.5 (±1.10) | 0.08 | 19 | 3.2 (±1.17) | 0.08 |
|  |  | **S3** | 10 | 3.1 (±1.29) | 15 | 3.7 (±0.96) | - | 8 | 3.0 (±0.93) | - |
|  |  | **S4** | 22 | 3.3 (±1.67) | 25 | 2.9 (±1.54) | -0.22 | 24 | 3.0 (±1.57) | -0.18 |
| **Providing feedback**  **(1-5)** | I give feedback to the children about their physical activity behaviour.  *For example, by discussing their physical activity behaviour and giving ideas for more ways of being physically active.* | **S1** | 26 | 3.6 (±0.90) | 24 | 3.6 (±0.71) | 0.23 | 29 | 4.2 (±0.97) | 0.85 |
|  |  | **S2** | 15 | 3.7 (±1.10) | 23 | 4.1 (±1.00) | 0.14 | 19 | 3.7 (±0.89) | 0.23 |
|  |  | **S3** | 10 | 3.7 (±1.16) | 15 | 4.1 (±0.59) | - | 8 | 4.0 (±0.76) | - |
|  |  | **S4** | 22 | 3.5 (±1.37) | 25 | 3.6 (±1.32) | 0.14 | 24 | 3.7 (±1.08) | 0.24 |
| **Instrumental feeding**  **(1-5)** | I reward the children sometimes by watching a movie together or letting them be on the computer when they did well.  *For example, when it is almost holiday and they worked well in the last weeks.* | **S1** | 24 | 3.6 (±1.44) | 24 | 3.2 (±1.18) | -0.21 | 29 | 4.0 (±1.24) | 0.62 |
|  |  | **S2** | 15 | 3.5 (±1.25) | 22 | 3.7 (±1.03) | 0.12 | 19 | 3.4 (±1.30) | -0.07 |
|  |  | **S3** | 10 | 4.4 (±1.08) | 15 | 4.0 (±1.13) | - | 8 | 3.8 (±1.58) | - |
|  |  | **S4** | 22 | 4.0 (±1.25) | 25 | 4.2 (±1.11) | 0.08 | 24 | 4.0 (±1.37) | -0.04 |
| **Pressure to be physically active**  **(1-5)** | I insist that the children be physically active.  *For example, by insisting that children always go outside during breaks, or that children always participate during PE or energizers.* | **S1** | 26 | 4.3 (±0.62) | 24 | 4.1 (±0.61) | -0.34 | 29 | 4.1 (±0.64) | -0.10 |
|  |  | **S2** | 15 | 4.5 (±0.83) | 23 | 4.5 (±0.59) | 0.09 | 19 | 4.4 (±0.50) | 0.10 |
|  |  | **S3** | 10 | 4.5 (±0.53) | 15 | 4.5 (±0.64) | - | 8 | 4.6 (±0.52) | - |
|  |  | **S4** | 22 | 4.2 (±1.01) | 25 | 4.3 (±0.85) | 0.19 | 24 | 4.5 (±0.78) | 0.31 |
| **Routines**  **(1-5)** | I ensure healthy PA habits in school.  *For example, by planning the times for energizers. By ensuring that the PA activity is fun and that everybody can join in.* | **S1** | 26 | 3.9 (±0.77) | 23 | 3.8 (±0.49) | 0.08 | 29 | 4.5 (±1.18) | 0.86 |
|  |  | **S2** | 15 | 4.1 (±0.80) | 23 | 4.1 (±0.55) | 0.21 | 19 | 4.1 (±0.78) | 0.00 |
|  |  | **S3** | 10 | 4.3 (±0.82) | 15 | 3.9 (±0.46) | - | 8 | 4.3 (±0.71) | - |
|  |  | **S4** | 22 | 3.8 (±1.15) | 25 | 4.4 (±0.91) | 0.57 | 24 | 4.4 (±0.71) | 0.62 |
| **Rules**  **(1-5)** | I strictly follow the school policy on physical activity in the school and in the classroom.  *For example, by strictly following the rules in the classroom or school yard regarding physical activity.* | **S1** | 26 | 4.2 (±0.98) | 24 | 4.1 (±0.58) | -0.09 | 29 | 4.3 (±0.67) | 0.27 |
|  |  | **S2** | 15 | 4.4 (±0.63) | 23 | 4.3 (±0.56) | 0.40 | 19 | 4.4 (±0.61) | 0.28 |
|  |  | **S3** | 10 | 4.5 (±0.53) | 15 | 4.5 (±0.74) | - | 8 | 4.6 (±0.74) | - |
|  |  | **S4** | 22 | 4.5 (±1.14) | 25 | 4.1 (±0.91) | -0.34 | 24 | 4.5 (±0.66) | 0.09 |
| **Warning PA**  **(1-5)** | I warn the children about the possible risks of physical activity and playing outside.  *For example, by warning the children they can hurt themselves or get dirty when playing outside.* | **S1** | 26 | 2.8 (±1.20) | 24 | 2.5 (±0.98) | -0.25 | 29 | 3.2 (±1.54) | 0.31 |
|  |  | **S2** | 15 | 2.8 (±0.94) | 22 | 3.3 (±1.36) | 0.60 | 19 | 2.7 (±1.45) | 0.00 |
|  |  | **S3** | 10 | 2.6 (±1.35) | 15 | 3.1 (±1.44) | - | 8 | 2.4 (±1.30) | - |
|  |  | **S4** | 22 | 3.1 (±1.68) | 25 | 2.6 (±1.35) | -0.28 | 24 | 3.1 (±1.64) | 0.00 |
| **Discour-agement**  **(1-5)** | I correct children when they do not sit quietly during lessons.  *For example, when a child is moving on his chair continuously.* | **S1** | 26 | 3.9 (±0.94) | 24 | 3.5 (±0.89) | -0.47 | 29 | 3.7 (±1.11) | -0.19 |
|  |  | **S2** | 15 | 3.4 (±1.30) | 22 | 3.5 (±0.96) | -0.40 | 19 | 3.8 (±0.83) | -0.30 |
|  |  | **S3** | 10 | 3.9 (±1.10) | 15 | 3.4 (±0.63) | - | 8 | 3.0 (±0.93) | - |
|  |  | **S4** | 22 | 4.4 (±0.85) | 25 | 3.9 (±0.93) | -0.47 | 24 | 3.9 (±1.06) | -0.57 |

**Standardized effect sizes (Cohen’s d) were calculated by: (mean at follow-up time of measurement minus mean at baseline, i.e. measurement prior to implementation of changes) divided by standard deviation at baseline. Only teachers who filled in both the questionnaire at baseline (T0) and at T1/T2 were included in this calculation.*

**Additional file 1b. Nutrition- and PA-related practices of parents**

|  | | | **T0** | | **T1** | | | **T2** | | | | |  |
| --- | --- | --- | --- | --- | --- | --- | --- | --- | --- | --- | --- | --- | --- |
|  |  |  | **N** | **Mean (±SD)** | **N** | **Mean (±SD)** | **Effect size*** | **N** | **Mean (±SD)** | | **Effect size*** | |  |
| **Nutrition-related practices** | | | | | | | | | | | | | |
| **Availability (1-5)** | I ensure healthy foods are available at home for my child.  *For example, by bringing vegetables and fruit into the house or by having enough choice in different healthy products for my child.* | **S1** | 132 | 4.3 (±0.67) | 109 | 4.4 (±0.60) | 0.14 | 111 | | 4.3 (±0.63) | | -0.09 |  |
|  |  | **S2** | 62 | 4.5 (±0.57) | 68 | 4.5 (±0.70) | 0.05 | 55 | | 4.5 (±0.63) | | 0.20 |  |
|  |  | **S3** | 80 | 4.3 (±0.77) | 69 | 4.5 (±0.56) | 0.20 | 69 | | 4.4 (±0.53) | | 0.21 |  |
|  |  | **S4** | 131 | 4.4 (±0.60) | 105 | 4.5 (±0.64) | 0.13 | 96 | | 4.5 (±0.58) | | 0.06 |  |
| **Encouragement (1-5)** | I encourage my child to eat healthily.  *For example, by encouraging my child to eat enough vegetables during the meal, by saying positive things about healthy products or by encouraging my child to eat many different types of food.* | **S1** | 132 | 4.4 (±0.64) | 109 | 4.4 (±0.64) | 0.04 | 111 | | 4.3 (±0.59) | | -0.12 |  |
|  |  | **S2** | 62 | 4.5 (±0.50) | 68 | 4.5 (±0.59) | 0.29 | 55 | | 4.5 (±0.54) | | 0.10 |  |
|  |  | **S3** | 80 | 4.3 (±0.67) | 69 | 4.5 (±0.50) | 0.18 | 69 | | 4.4 (±0.50) | | 0.14 |  |
|  |  | **S4** | 131 | 4.4 (±0.63) | 105 | 4.5 (±0.56) | 0.17 | 96 | | 4.4 (±0.54) | | 0.03 |  |
| **Healthy modelling**  **(1-5)** | I consciously eat healthy foods when my child is around.  *For example, by eating fruit when my child is around or by being enthusiastic about healthy food when my child is around.* | **S1** | 132 | 4.1 (±0.86) | 109 | 4.0 (±0.88) | -0.04 | 111 | | 3.9 (±0.78) | | -0.17 |  |
|  |  | **S2** | 62 | 4.0 (±0.76) | 68 | 4.0 (±0.83) | 0.12 | 55 | | 4.1 (±0.75) | | 0.00 |  |
|  |  | **S3** | 80 | 4.0 (±0.81) | 69 | 3.9 (±0.89) | 0.00 | 69 | | 3.9 (±0.65) | | 0.13 |  |
|  |  | **S4** | 131 | 4.1 (±0.82) | 105 | 4.2 (±0.75) | 0.06 | 96 | | 4.0 (±0.83) | | -0.07 |  |
| **Educating**  **(1-5)** | I teach my child about nutrition.  *For example, by explaining that eating something healthy is better than snacking, or that drinking soft drinks and eating snacks is not good for their teeth or that you can gain weight from it.* | **S1** | 132 | 4.2 (±0.65) | 109 | 4.1 (±0.68) | -0.08 | 111 | | 4.1 (±0.62) | | -0.08 |  |
|  |  | **S2** | 62 | 4.4 (±0.59) | 68 | 4.3 (±0.80) | -0.05 | 55 | | 4.3 (±0.70) | | -0.33 |  |
|  |  | **S3** | 80 | 4.2 (±0.70) | 69 | 4.2 (±0.64) | 0.12 | 69 | | 4.2 (±0.70) | | 0.00 |  |
|  |  | **S4** | 131 | 4.3 (±0.66) | 105 | 4.3 (±0.69) | 0.02 | 96 | | 4.2 (±0.65) | | -0.18 |  |
| **Involving**  **(1-5)** | I involve my child in things that concern eating/drinking.  *For example, by involving my child in shopping, having dinner chosen, or helping to prepare food or to lay the table.* | **S1** | 132 | 4.1 (±0.76) | 109 | 4.2 (±0.70) | 0.14 | 111 | | 4.2 (±0.65) | | 0.06 |  |
|  |  | **S2** | 62 | 4.0 (±0.71) | 68 | 4.0 (±0.87) | -0.20 | 55 | | 4.1 (±0.73) | | -0.06 |  |
|  |  | **S3** | 80 | 4.0 (±0.84) | 69 | 4.2 (±0.69) | 0.14 | 69 | | 4.0 (±0.65) | | -0.06 |  |
|  |  | **S4** | 131 | 4.2 (±0.63) | 105 | 4.2 (±0.76) | -0.13 | 96 | | 4.1 (±0.76) | | 0.00 |  |
| **Emotional feeding (1-5)** | I give my child unhealthy foods to make him/her feel better.  *For example, by comforting or calming my child with sweets or snacks if my child has pain or by giving sweets or snacks to cheer up my child.* | **S1** | 132 | 1.9 (±1.07) | 109 | 1.6 (±0.86) | -0.26 | 111 | | 1.8 (±1.03) | | -0.06 |  |
|  |  | **S2** | 62 | 1.6 (±0.86) | 68 | 1.6 (±0.88) | 0.00 | 55 | | 1.5 (±0.84) | | -0.40 |  |
|  |  | **S3** | 80 | 1.7 (±0.87) | 69 | 1.6 (±0.90) | -0.10 | 69 | | 1.7 (±0.85) | | 0.17 |  |
|  |  | **S4** | 131 | 1.7 (±0.94) | 105 | 1.7 (±0.98) | 0.12 | 96 | | 1.6 (±0.83) | | -0.11 |  |
| **Rules**  **(1-5)** | I have rules for my child concerning unhealthy foods.  *For example, by having the rule that my child first has to ask if it is a soft drink, that my child is not allowed to eat sweets and snacks just before the meal or that it does not get certain kinds of sweets.* | **S1** | 132 | 4.1 (±0.90) | 109 | 4.0 (±0.92) | -0.11 | 111 | | 4.1 (±0.83) | | 0.11 |  |
|  |  | **S2** | 62 | 4.2 (±0.91) | 68 | 4.2 (±0.97) | 0.18 | 55 | | 3.9 (±1.03) | | 0.28 |  |
|  |  | **S3** | 80 | 4.0 (±0.81) | 69 | 4.1 (±0.89) | 0.23 | 69 | | 3.9 (±0.93) | | -0.09 |  |
|  |  | **S4** | 131 | 4.3 (±0.75) | 105 | 4.3 (±0.78) | -0.22 | 96 | | 4.1 (±1.01) | | -0.16 |  |
| **Structure**  **(1-5)** | I create structure concerning unhealthy products.  *For example, by giving my child only sweets and snacks at fixed times, such as when my child comes out of school.* | **S1** | 132 | 3.7 (±1.07) | 109 | 3.9 (±0.84) | 0.14 | 111 | | 3.6 (±0.93) | | 0.10 |  |
|  |  | **S2** | 62 | 3.7 (±1.04) | 68 | 3.6 (±1.10) | -0.09 | 55 | | 3.9 (±0.92) | | 0.22 |  |
|  |  | **S3** | 80 | 3.6 (±0.94) | 69 | 3.7 (±0.88) | 0.23 | 69 | | 3.7 (±0.89) | | 0.36 |  |
|  |  | **S4** | 131 | 3.9 (±0.88) | 105 | 4.0 (±0.87) | -0.10 | 96 | | 3.8 (±0.97) | | -0.23 |  |
| **Accessibility**  **(1-5)** | I ensure my child has access to unhealthy products.  *For example, by storing sweets and snacks in a place that my child can access.* | **S1** | 132 | 2.5 (±1.09) | 109 | 2.3 (±1.13) | -0.11 | 111 | | 2.4 (±1.08) | | 0.13 |  |
|  |  | **S2** | 62 | 2.5 (±0.99) | 68 | 2.5 (±1.13) | 0.00 | 55 | | 2.4 (±1.08) | | -0.15 |  |
|  |  | **S3** | 80 | 2.5 (±0.94) | 69 | 2.3 (±0.95) | -0.16 | 69 | | 2.1 (±0.97) | | -0.23 |  |
|  |  | **S4** | 131 | 2.2 (±0.95) | 105 | 2.2 (±1.07) | 0.03 | 96 | | 2.2 (±1.19) | | 0.08 |  |
| **PA-related practices** | | | | | | | | | | | | | |
| **Rules PA**  **(1-5)** | I have rules for my child about being physically active.  *For example, by making arrangements that my child always goes to school by bike or goes to the sports club every week.* | **S1** | 134 | 3.7 (±1.14) | 111 | 3.5 (±1.25) | -0.27 | 112 | | 3.5 (±1.16) | | 0.03 |  |
|  |  | **S2** | 67 | 3.8 (±1.24) | 71 | 4.0 (±0.95) | 0.07 | 57 | | 3.7 (±1.06) | | 0.26 |  |
|  |  | **S3** | 82 | 3.6 (±1.22) | 70 | 3.7 (±1.22) | 0.13 | 71 | | 3.5 (±1.26) | | 0.23 |  |
|  |  | **S4** | 136 | 3.7 (±1.10) | 108 | 3.8 (±1.14) | -0.01 | 103 | | 3.9 (±1.15) | | 0.10 |  |
| **Accessibility PA**  **(1-5)** | I ensure my child has access to places to be physically active.  *For example, by bringing my child to PA. Or by taking my child to the park, the woods or a playing field.* | **S1** | 134 | 4.2 (±0.78) | 111 | 4.3 (±0.58) | 0.03 | 112 | | 4.2 (±0.60) | | -0.04 |  |
|  |  | **S2** | 67 | 4.3 (±0.90) | 71 | 4.3 (±0.79) | 0.03 | 57 | | 4.4 (±0.60) | | 0.13 |  |
|  |  | **S3** | 82 | 4.2 (±0.84) | 70 | 4.4 (±0.70) | 0.11 | 71 | | 4.3 (±0.55) | | -0.03 |  |
|  |  | **S4** | 136 | 4.4 (±0.66) | 108 | 4.3 (±0.75) | -0.25 | 103 | | 4.4 (±0.63) | | -0.14 |  |
| **Encouragement**  **(1-5)** | I encourage my child to be physically active.  *For example, by naming positive aspects about movement.* | **S1** | 134 | 4.3 (±0.74) | 111 | 4.2 (±0.71) | -0.16 | 112 | | 4.1 (±0.66) | | -0.35 |  |
|  |  | **S2** | 67 | 4.3 (±0.86) | 71 | 4.3 (±0.75) | -0.13 | 57 | | 4.3 (±0.63) | | 0.00 |  |
|  |  | **S3** | 82 | 4.2 (±0.86) | 70 | 4.2 (±0.73) | 0.06 | 71 | | 4.4 (±0.52) | | 0.23 |  |
|  |  | **S4** | 136 | 4.2 (±0.80) | 108 | 4.4 (±0.67) | 0.00 | 103 | | 4.3 (±0.60) | | 0.02 |  |
| **Availability PA**  **(1-5)** | I ensure PA-friendly equipment is available for my child.  *For example, by having fun PA games or outdoor toys such as a skipping rope or football.* | **S1** | 134 | 4.3 (±0.73) | 111 | 4.3 (±0.69) | 0.02 | 112 | | 4.1 (±0.70) | | -0.09 |  |
|  |  | **S2** | 67 | 4.4 (±0.84) | 71 | 4.3 (±0.66) | -0.41 | 57 | | 4.3 (±0.64) | | -0.08 |  |
|  |  | **S3** | 82 | 4.2 (±0.84) | 70 | 4.2 (±0.73) | -0.22 | 71 | | 4.3 (±0.56) | | 0.00 |  |
|  |  | **S4** | 136 | 4.3 (±0.68) | 108 | 4.3 (±0.73) | -0.06 | 103 | | 4.3 (±0.59) | | -0.48 |  |
| **Educating**  **(1-5)** | I teach my child about physical activity.  *For example, by teaching my child new ways of moving, such as exercise is good for health, or by talking about sports and its rules.* | **S1** | 134 | 3.9 (±0.84) | 111 | 3.8 (±0.95) | -0.08 | 112 | | 3.9 (±0.78) | | 0.05 |  |
|  |  | **S2** | 67 | 4.0 (±0.84) | 71 | 4.0 (±0.84) | 0.17 | 57 | | 4.0 (±0.71) | | 0.36 |  |
|  |  | **S3** | 82 | 3.9 (±0.78) | 70 | 4.0 (±0.86) | 0.24 | 71 | | 4.1 (±0.79) | | 0.35 |  |
|  |  | **S4** | 136 | 3.9 (±0.92) | 108 | 4.0 (±0.81) | 0.01 | 103 | | 3.9 (±0.73) | | -0.05 |  |
| **Involving**  **(1-5)** | I involve my child in physical activity.  *For example, by playing outside together, exercising, walking, dancing or playing an active game (such as a ball game). Or by letting my child help me with gardening.* | **S1** | 134 | 4.2 (±0.71) | 111 | 4.1 (±0.77) | -0.25 | 112 | | 4.1 (±0.70) | | -0.33 |  |
|  |  | **S2** | 67 | 4.0 (±0.91) | 71 | 4.0 (±0.86) | -0.18 | 57 | | 4.0 (±0.79) | | -0.26 |  |
|  |  | **S3** | 82 | 4.1 (±0.76) | 70 | 4.1 (±0.71) | -0.07 | 71 | | 4.1 (±0.63) | | 0.04 |  |
|  |  | **S4** | 136 | 4.2 (±0.70) | 108 | 4.2 (±0.79) | -0.15 | 103 | | 4.1 (±0.67) | | -0.21 |  |
| **Routines PA**  **(1-5)** | I ensure my child acquires good physical activity habits.  *For example, by bicycling or going on foot as much as possible instead of taking the car.* | **S1** | 134 | 3.8 (±0.93) | 111 | 3.8 (±0.97) | -0.04 | 112 | | 3.7 (±0.97) | | 0.03 |  |
|  |  | **S2** | 67 | 3.6 (±1.02) | 71 | 3.7 (±0.97) | -0.03 | 57 | | 3.7 (±0.96) | | 0.27 |  |
|  |  | **S3** | 82 | 3.7 (±0.86) | 70 | 3.8 (±0.98) | 0.08 | 71 | | 3.8 (±0.78) | | 0.29 |  |
|  |  | **S4** | 136 | 3.7 (±0.94) | 108 | 3.9 (±0.88) | 0.11 | 103 | | 3.7 (±0.92) | | 0.00 |  |
| **Healthy modelling PA**  **(1-5)** | I ensure my child sees me being physically active.  *For example, that my child sees me walking, exercising or gardening.* | **S1** | 134 | 4.1 (±0.77) | 111 | 4.0 (±0.85) | 0.03 | 112 | | 4.0 (±0.81) | | -0.09 |  |
|  |  | **S2** | 67 | 3.9 (±0.99) | 71 | 3.9 (±0.92) | -0.16 | 57 | | 4.1 (±0.81) | | 0.21 |  |
|  |  | **S3** | 82 | 3.9 (±0.83) | 70 | 4.0 (±0.87) | 0.18 | 71 | | 4.1 (±0.65) | | 0.07 |  |
|  |  | **S4** | 136 | 3.9 (±0.82) | 108 | 4.0 (±0.80) | 0.10 | 103 | | 3.9 (±0.82) | | -0.21 |  |
| **Warning**  **(1-5)** | I warn my child about the risks of being physical active and playing outside.  *For example, by saying to my child that s/he can be hurt or get dirty. Or by expressing my concerns about possible dangers, strange people or traffic safety.* | **S1** | 134 | 3.8 (±1.09) | 111 | 3.8 (±1.01) | -0.06 | 112 | | 3.7 (±0.94) | | -0.15 |  |
|  |  | **S2** | 67 | 3.8 (±1.12) | 71 | 3.8 (±1.15) | -0.12 | 57 | | 3.8 (±1.09) | | -0.38 |  |
|  |  | **S3** | 82 | 3.8 (±1.16) | 70 | 3.7 (±1.15) | -0.15 | 71 | | 3.6 (±1.14) | | -0.18 |  |
|  |  | **S4** | 136 | 3.6 (±1.13) | 108 | 3.7 (±1.09) | 0.06 | 103 | | 3.5 (±1.20) | | -0.25 |  |
| **Emotional feeding TV**  **(1-5)** | I allow my child to be on the computer or watch TV to make him/her feel better.  *For example, by comforting, calming my child by having him / her watch TV or playing a game on a laptop or tablet.* | **S1** | 134 | 2.2 (±1.01) | 110 | 2.2 (±0.92) | 0.04 | 112 | | 2.3 (±0.96) | | 0.14 |  |
|  |  | **S2** | 66 | 2.2 (±0.88) | 70 | 2.3 (±1.02) | -0.03 | 56 | | 2.1 (±0.86) | | -0.31 |  |
|  |  | **S3** | 82 | 2.5 (±1.03) | 69 | 2.2 (±1.03) | -0.26 | 71 | | 2.2 (±1.00) | | -0.33 |  |
|  |  | **S4** | 136 | 2.3 (±0.98) | 108 | 2.3 (±0.96) | 0.09 | 100 | | 2.2 (±1.05) | | -0.13 |  |
| **Healthy modelling TV**  **(1-5)** | I consciously do not use the computer or watch TV when my child is around.  *For example, by using a computer or watching TV when my child is already in bed.* | **S1** | 134 | 2.5 (±0.99) | 110 | 2.5 (±0.96) | 0.09 | 112 | | 2.6 (±0.91) | | 0.10 |  |
|  |  | **S2** | 66 | 2.2 (±0.81) | 70 | 2.5 (±0.94) | 0.14 | 56 | | 2.6 (±1.06) | | 0.00 |  |
|  |  | **S3** | 82 | 2.5 (±0.95) | 69 | 2.5 (±0.92) | -0.19 | 71 | | 2.5 (±0.81) | | -0.13 |  |
|  |  | **S4** | 136 | 2.6 (±0.96) | 108 | 2.5 (±0.90) | -0.02 | 100 | | 2.5 (±1.09) | | -0.11 |  |
| **Rules TV**  **(1-5)** | I have rules for my child concerning the use of the computer/TV.  *For example, by setting limits for how long my child can watch TV.* | **S1** | 134 | 3.8 (±0.95) | 110 | 3.9 (±0.95) | 0.03 | 112 | | 3.9 (±0.95) | | 0.00 |  |
|  |  | **S2** | 66 | 3.6 (±1.12) | 70 | 3.9 (±0.83) | 0.13 | 56 | | 3.9 (±0.88) | | 0.16 |  |
|  |  | **S3** | 82 | 3.8 (±0.94) | 69 | 3.9 (±0.83) | 0.21 | 71 | | 3.7 (±1.04) | | 0.05 |  |
|  |  | **S4** | 136 | 3.9 (±0.90) | 108 | 3.9 (±0.82) | 0.03 | 100 | | 3.8 (±0.96) | | -0.04 |  |
| **Structure TV**  **(1-5)** | I create structure concerning the use of the computer/TV.  *For example, by limiting times when my child can watch TV or use the computer or laptop.* | **S1** | 134 | 3.7 (±0.95) | 110 | 3.8 (±0.99) | 0.03 | 112 | | 3.8 (±0.97) | | 0.03 |  |
|  |  | **S2** | 66 | 3.4 (±1.08) | 70 | 3.6 (±0.92) | -0.06 | 56 | | 3.7 (±0.86) | | -0.06 |  |
|  |  | **S3** | 82 | 3.6 (±0.87) | 69 | 3.7 (±0.98) | -0.03 | 71 | | 3.6 (±1.08) | | -0.03 |  |
|  |  | **S4** | 136 | 3.8 (±0.87) | 108 | 3.8 (±0.89) | -0.04 | 100 | | 3.7 (±0.97) | | -0.04 |  |
| **Accessibility TV**  **(1-5)** | I ensure my child has access to the computer/TV/tablet.  *For example, by always having the television on and having the laptop or tablet on the table.* | **S1** | 134 | 2.6 (±0.95) | 110 | 2.8 (±0.94) | 0.31 | 112 | | 2.8 (±1.03) | | 0.44 |  |
|  |  | **S2** | 66 | 2.6 (±1.09) | 70 | 2.9 (±1.03) | 0.23 | 56 | | 2.8 (±1.06) | | 0.24 |  |
|  |  | **S3** | 82 | 2.8 (±0.97) | 69 | 2.5 (±0.92) | -0.16 | 71 | | 2.8 (±0.91) | | 0.02 |  |
|  |  | **S4** | 136 | 2.6 (±1.02) | 108 | 2.6 (±1.05) | 0.07 | 100 | | 2.6 (±0.99) | | 0.00 |  |

**Standardized effect sizes (Cohen’s d) were calculated by: (mean at follow-up time of measurement minus mean at baseline, i.e. measurement prior to implementation of changes) divided by standard deviation at baseline. Only parents who filled in both the questionnaire at baseline (T0) and at T1/T2 were included in this calculation.*
