# Supplementary material for: Process evaluation of the healthy primary School of the Future: the key learning points
Source: BMC Public Health. 2019 Jun 6;19:698. doi: 10.1186/s12889-019-6947-2 (PMC6554901; doi:10.1186/s12889-019-6947-2)
Supplement: Supplementary file 2 — Perceived potential barriers for HPSF. Description: Presence of potential barriers for HPSF according to teachers and external pedagogical employees (DOCX 83 kb) [file 12889_2019_6947_MOESM2_ESM.docx]

**Additional file 2. Perceived potential barriers for HPSF**

**Additional file 2a. Presence of potential barriers for HPSF according to teachers**

| **Potential barriers (1-10)** |  | | **Nov ‘15** | | | **May ‘16** | | | | **Nov ‘16** | | **May ‘17** | | **Nov ‘17** | | |
| --- | --- | --- | --- | --- | --- | --- | --- | --- | --- | --- | --- | --- | --- | --- | --- | --- |
|  |  |  | **N** | **Mean (±SD)** | | **N** | | **Mean (±SD)** | | **N** | **Mean (±SD)** | **N** | **Mean (±SD)** | **N** | **Mean (±SD)** | |
| **Implementers: teachers** | | | | | | | | | | | | | | | | |
| **Attitude: I like to implement the activities** | **S1** | 11 | | | 7.5 (±1.21) | | 9 | | 7.6 (±1.33) | 9 | 8.0 (±0.50) | 3 | 8.0 (±0.00) | 12 | | 6.8 (±1.40) |
|  | **S2** | 10 | | | 8.4 (±0.70) | | 6 | | 9.2 (±0.98) | 15 | 8.4 (±1.40) | 19 | 8.4 (±1.12) | 12 | | 8.2 (±1.19) |
|  | **S3** | 9 | | | 7.9 (±1.05) | | 3 | | 8.0 (±0.00) | 7 | 8.3 (±0.76) | 6 | 8.8 (±0.98) | 6 | | 8.8 (±0.98) |
|  | **S4** | 14 | | | 7.9 (±0.77) | | 8 | | 7.6 (±0.52) | 15 | 8.2 (±0.65) | 14 | 8.0 (±1.11) | 12 | | 8.5 (±0.78) |
| ***Self-efficacy:* I am able to implement the activities.** | **S1** | 11 | | | 7.5 (±0.69) | | 9 | | 7.1 (±1.54) | 9 | 7.7 (±0.50) | 3 | 7.7 (±0.58) | 12 | | 7.1 (±1.78) |
|  | **S2** | 10 | | | 8.3 (±0.82) | | 6 | | 8.3 (±1.21) | 15 | 8.0 (±1.36) | 19 | 7.9 (±1.05) | 11 | | 7.9 (±1.45) |
|  | **S3** | 9 | | | 7.4 (±1.33) | | 3 | | 7.3 (±0.58) | 7 | 7.6 (±0.53) | 6 | 8.5 (±1.52) | 6 | | 8.7 (±1.03) |
|  | **S4** | 13 | | | 7.9 (±0.76) | | 8 | | 7.5 (±0.76) | 14 | 7.7 (±0.99) | 13 | 7.8 (±0.99) | 12 | | 8.4 (±0.79) |
| ***Motivation*: I am motivated to implement the activities.** | **S1** | 11 | | | 8.0 (±0.77) | | 9 | | 7.7 (±1.22) | 9 | 8.1 (±0.78) | 3 | 8.3 (±0.58) | 12 | | 7.2 (±1.19) |
|  | **S2** | 10 | | | 8.6 (±0.70) | | 6 | | 9.2 (±0.98) | 15 | 8.9 (±1.06) | 19 | 8.5 (±1.12) | 12 | | 8.5 (±1.00) |
|  | **S3** | 9 | | | 8.3 (±1.12) | | 3 | | 8.0 (±0.00) | 7 | 8.7 (±0.76) | 6 | 8.8 (±0.98) | 6 | | 8.7 (±1.03) |
|  | **S4** | 13 | | | 8.2 (±0.90) | | 8 | | 7.8 (±0.71) | 14 | 8.3 (±0.58) | 14 | 8.1 (±1.00) | 12 | | 8.4 (±0.63) |
| ***Knowledge*: I have enough knowledge to implement the activities.** | **S1** | 11 | | | 7.5 (±0.52) | | 9 | | 8.0 (±1.32) | 8 | 7.9 (±0.64) | 4 | 8.5 (±0.58) | 12 | | 8.2 (±0.94) |
|  | **S2** | 10 | | | 8.3 (±0.82) | | 5 | | 8.8 (±0.84) | 15 | 8.2 (±1.01) | 18 | 8.0 (±1.14) | 12 | | 8.4 (±1.08) |
|  | **S3** | 10 | | | 7.3 (±1.42) | | 3 | | 7.3 (±0.58) | 7 | 8.1 (±1.07) | 6 | 8.3 (±0.99) | 6 | | 8.7 (±1.03) |
|  | **S4** | 13 | | | 7.8 (±0.99) | | 8 | | 7.8 (±0.71) | 14 | 8.1 (±0.62) | 14 | 7.9 (±0.92) | 12 | | 8.5 (±0.80) |
| ***Skills:* I have enough skills to implement the activities.** | **S1** | 11 | | | 7.7 (±0.47) | | 9 | | 8.0 (±1.32) | 8 | 8.1 (±0.64) | 4 | 8.5 (±0.58) | 12 | | 8.4 (±1.00) |
|  | **S2** | 10 | | | 8.4 (±1.07) | | 5 | | 9.2 (±0.84) | 14 | 8.2 (±1.12) | 18 | 8.3 (±1.14) | 12 | | 8.8 (±0.97) |
|  | **S3** | 10 | | | 7.8 (±1.40) | | 3 | | 7.3 (±0.58) | 7 | 7.9 (±1.07) | 6 | 8.2 (±0.75) | 6 | | 8.7 (±1.03) |
|  | **S4** | 13 | | | 7.9 (±1.04) | | 9 | | 8.0 (±1.00) | 14 | 8.2 (±0.89) | 14 | 8.1 (±0.86) | 11 | | 8.4 (±0.67) |
| ***Modelling behavior*: I try to give the good example to children concerning healthy dietary and PA behaviours.** | **S1** | 14 | | | 8.2 (±0.80) | | 14 | | 8.9 (±0.66) | 16 | 8.4 (±1.09) | 6 | 8.7 (±0.52) | 16 | | 8.1 (±0.93) |
|  | **S2** | 10 | | | 9.0 (±0.82) | | 7 | | 8.9 (±1.22) | 17 | 9.1 (±0.78) | 20 | 8.6 (±1.10) | 18 | | 8.5 (±0.92) |
|  | **S3** | 10 | | | 8.5 (±1.08) | | 8 | | 8.6 (±0.92) | 8 | 8.3 (±1.28) | 6 | 8.7 (±0.82) | 9 | | 9.1 (±1.17) |
|  | **S4** | 17 | | | 7.5 (±1.28) | | 19 | | 7.6 (±0.92) | 19 | 7.5 (±1.54) | 19 | 8.1 (±1.04) | 12 | | 8.4 (±1.08) |
| ***Satisfaction:* The children like the activities in my opinion.** | **S1** | 13 | | | 7.7 (±0.75) | | 12 | | 8.1 (±0.79) | 14 | 8.2 (±0.98) | 4 | 8.5 (±0.58) | 15 | | 7.8 (±1.42) |
|  | **S2** | 10 | | | 8.1 (±0.88) | | 6 | | 8.8 (±0.98) | 15 | 8.7 (±1.22) | 20 | 8.0 (±0.92) | 17 | | 7.9 (±0.75) |
|  | **S3** | 10 | | | 8.3 (±1.25) | | 8 | | 8.4 (±0.92) | 8 | 8.5 (±1.07) | 6 | 8.3 (±1.03) | 9 | | 8.6 (±0.88) |
|  | **S4** | 17 | | | 8.1 (±1.11) | | 18 | | 7.4 (±1.76) | 19 | 8.6 (±0.77) | 18 | 8.3 (±1.19) | 12 | | 8.8 (±0.94) |
| ***Cooperation:* The children cooperate well with the activities in my opinion.** | **S1** | 13 | | | 7.2 (±0.60) | | 12 | | 8.0 (±0.95) | 15 | 7.8 (±1.15) | 4 | 8.5 (±0.58) | 15 | | 7.5 (±1.46) |
|  | **S2** | 10 | | | 8.1 (±1.10) | | 6 | | 8.7 (±0.82) | 17 | 8.7 (±1.10) | 20 | 8.0 (±0.89) | 18 | | 7.8 (±1.00) |
|  | **S3** | 10 | | | 8.4 (±1.26) | | 8 | | 8.3 (±0.89) | 8 | 8.3 (±0.71) | 6 | 8.3 (±1.03) | 9 | | 8.3 (±1.00) |
|  | **S4** | 17 | | | 8.2 (±0.83) | | 19 | | 7.4 (±1.77) | 19 | 8.3 (±0.93) | 18 | 8.1 (±1.08) | 12 | | 8.6 (±1.08) |
| ***Perceived outcome importance*: Children have healthier dietary and PA behaviours due to the activities in my opinion.** | **S1** | 13 | | | 7.8 (±0.80) | | 12 | | 7.6 (±1.16) | 14 | 8.0 (±0.96) | 3 | 8.7 (±0.58) | 15 | | 7.0 (±1.07) |
|  | **S2** | 10 | | | 8.6 (±0.97) | | 6 | | 9.0 (±0.89) | 18 | 8.4 (±1.24) | 20 | 8.0 (±0.92) | 18 | | 7.9 (±1.24) |
|  | **S3** | 10 | | | 7.5 (±1.72) | | 8 | | 7.6 (±1.51) | 8 | 7.9 (±0.99) | 6 | 7.8 (±1.47) | 9 | | 8.0 (±1.12) |
|  | **S4** | 17 | | | 6.5 (±1.46) | | 19 | | 6.3 (±1.37) | 19 | 6.8 (±1.01) | 19 | 6.9 (±1.15) | 12 | | 6.1 (±1.73) |
| ***Personal advantage:* I see also other advantages of the activities.** | **S1** | 13 | | | 7.4 (±0.87) | | 10 | | 6.6 (±1.08) | 13 | 7.4 (±1.19) | 4 | 7.3 (±0.96) | 15 | | 6.7 (±1.53) |
|  | **S2** | 10 | | | 7.7 (±1.29) | | 6 | | 8.0 (±1.26) | 18 | 8.1 (±2.00) | 19 | 7.6 (±1.26) | 17 | | 7.7 (±1.31) |
|  | **S3** | 10 | | | 8.1 (±0.99) | | 8 | | 7.4 (±1.06) | 8 | 7.9 (±0.99) | 5 | 8.6 (±1.52) | 9 | | 8.6 (±1.01) |
|  | **S4** | 17 | | | 8.0 (±0.84) | | 19 | | 7.5 (±1.74) | 19 | 8.2 (±0.98) | 18 | 7.6 (±1.09) | 12 | | 8.1 (±1.24) |
| ***Identification with organization:* I feel involved with the school I am working at.** | **S1** | 14 | | | 9.1 (±0.83) | | 14 | | 9.2 (±0.89) | 16 | 8.4 (±0.96) | 6 | 8.5 (±0.55) | 16 | | 8.1 (±0.85) |
|  | **S2** | 10 | | | 9.2 (±0.79) | | 7 | | 9.7 (±0.49) | 17 | 8.9 (±1.32) | 21 | 8.9 (±1.04) | 18 | | 9.1 (±0.87) |
|  | **S3** | 10 | | | 9.2 (±0.92) | | 8 | | 8.9 (±0.99) | 8 | 8.4 (±1.19) | 5 | 8.2 (±2.17) | 9 | | 8.7 (±0.87) |
|  | **S4** | 17 | | | 9.0 (±0.79) | | 19 | | 8.9 (±0.74) | 18 | 8.1 (±1.17) | 18 | 8.3 (±0.67) | 12 | | 8.6 (±1.16) |
| ***Perceived outcome feasibility*: It is important for children to have healthier dietary and PA behaviours in my opinion.** | **S1** | 14 | | | 8.9 (±0.86) | | 14 | | 9.2 (±0.89) | 16 | 8.9 (±0.89) | 6 | 9.0 (±0.63) | 16 | | 9.2 (±0.83) |
|  | **S2** | 10 | | | 9.2 (±0.79) | | 7 | | 9.7 (±0.49) | 17 | 9.3 (±0.77) | 21 | 9.4 (±0.74) | 18 | | 9.3 (±0.83) |
|  | **S3** | 10 | | | 8.8 (±1.03) | | 8 | | 9.4 (±0.74) | 8 | 8.8 (±1.28) | 6 | 9.0 (±0.89) | 9 | | 9.4 (±0.73) |
|  | **S4** | 17 | | | 8.6 (±0.61) | | 19 | | 8.8 (±0.79) | 19 | 8.9 (±0.91) | 19 | 8.9 (±0.91) | 12 | | 9.0 (±1.18) |
| ***Perceived task responsibility:* It is one of school’s responsibilities to encourage healthier dietary and PA behaviours of children in my opinion.** | **S1** | 14 | | | 7.4 (±1.28) | | 14 | | 7.1 (±1.61) | 16 | 7.0 (±1.49) | 6 | 7.0 (±1.41) | 16 | | 6.0 (±2.16) |
|  | **S2** | 10 | | | 8.6 (±0.97) | | 7 | | 9.4 (±0.63) | 16 | 7.9 (±1.89) | 21 | 7.8 (±1.41) | 18 | | 8.3 (±1.37) |
|  | **S3** | 10 | | | 7.6 (±1.26) | | 8 | | 7.5 (±1.41) | 8 | 8.1 (±1.81) | 6 | 8.3 (±1.63) | 9 | | 8.3 (±1.32) |
|  | **S4** | 17 | | | 7.1 (±0.99) | | 19 | | 6.7 (±1.28) | 19 | 6.7 (±2.00) | 19 | 7.1 (±1.70) | 12 | | 6.9 (±1.00) |
| **Innovation: HPSF** | | | | | | | | | | | | | | | | |
| *****Required time:* In my opinion it costs a lot of time to implement the activities.** | **S1** | 12 | | **4.9 (±2.02)** | | 8 | | 6.3 (±2.31) | | 9 | **4.3 (±2.45)** | 4 | **3.5 (±1.91)** | 12 | **4.9 (±2.02)** | |
|  | **S2** | 10 | | **5.4 (±2.12)** | | 5 | | 6.4 (±1.95) | | 15 | **5.1 (±2.77)** | 18 | **4.8 (±2.04)** | 12 | **5.8 (±2.53)** | |
|  | **S3** | 9 | | 6.9 (±2.57) | | 4 | | **5.5 (±2.52)** | | 8 | 7.0 (±2.51) | 5 | 7.6 (±2.19) | 6 | 8.0 (±2.28) | |
|  | **S4** | 14 | | 6.9 (±1.51) | | 9 | | **5.3 (±2.18)** | | 12 | **5.8 (±2.29)** | 14 | 6.1 (±2.23) | 11 | 6.5 (±2.34) | |
| *****Complexity:* In my opinion the activities are complex to implement.** | **S1** | 11 | | 6.7 (±2.49) | | 10 | | **1.8 (±1.32)** | | 9 | 8.1 (±1.90) | 3 | 8.7 (±2.31) | 12 | 8.8 (±2.04) | |
|  | **S2** | 9 | | 7.9 (±2.32) | | 5 | | 7.4 (±2.70) | | 14 | 8.4 (±1.91) | 19 | 7.4 (±2.99) | 12 | 8.0 (±2.49) | |
|  | **S3** | 9 | | 7.3 (±2.35) | | 3 | | **5.3 (±2.89)** | | 7 | 7.9 (±2.73) | 6 | 8.3 (±1.63) | 6 | 8.0 (±2.90) | |
|  | **S4** | 13 | | 7.8 (±1.30) | | 10 | | **3.4 (±1.96)** | | 13 | 7.8 (±2.08) | 12 | 8.3 (±1.76) | 12 | 8.0 (±2.09) | |
| ***Relevance:* In my opinion the activities are suitable for children.** | **S1** | 13 | | 7.4 (±1.04) | | 12 | | 7.6 (±1.07) | | 12 | 8.4 (±0.67) | 4 | 7.8 (±0.50) | 14 | 7.9 (±1.44) | |
|  | **S2** | 10 | | 8.0 (±0.94) | | 5 | | 8.4 (±1.34) | | 15 | 8.2 (±1.26) | 20 | 7.9 (±0.81) | 17 | 7.4 (±1.18) | |
|  | **S3** | 10 | | 8.2 (±0.79) | | 8 | | 8.5 (±0.93) | | 8 | 8.5 (±0.93) | 6 | 8.7 (±0.82) | 9 | 8.6 (±0.88) | |
|  | **S4** | 15 | | 7.7 (±0.94) | | 18 | | 7.8 (±0.99) | | 19 | 8.5 (±0.84) | 18 | 8.4 (±0.92) | 12 | 8.4 (±0.67) | |
| ***Observability*: In my opinion the effects of the activities are clearly visible.** | **S1** | 12 | | **5.9 (±1.51)** | | 12 | | **5.8 (±1.95)** | | 12 | 6.6 (±1.51) | 4 | 7.5 (±0.58) | 15 | **5.7 (±1.87)** | |
|  | **S2** | 10 | | 6.8 (±1.03) | | 6 | | 8.2 (±1.17) | | 18 | 7.8 (±1.20) | 20 | 7.2 (±1.04) | 18 | 7.2 (±1.11) | |
|  | **S3** | 10 | | 7.3 (±1.57) | | 8 | | 6.8 (±1.17) | | 8 | 7.3 (±0.89) | 6 | 7.3 (±1.75) | 9 | 7.7 (±1.50) | |
|  | **S4** | 17 | | 6.6 (±1.06) | | 18 | | 6.8 (±1.77) | | 19 | 7.1 (±1.18) | 19 | 6.7 (±1.69) | 12 | 7.3 (±1.60) | |
| ***Flexibility*: In my opinion the activities are flexible enough to work with.** | **S1** | 12 | | 6.5 (±1.51) | | 10 | | 6.7 (±1.70) | | 12 | 7.5 (±1.00) | 4 | 7.8 (±0.50) | 13 | 7.2 (±1.79) | |
|  | **S2** | 10 | | 7.6 (±0.76) | | 6 | | 7.0 (±3.03) | | 15 | 7.7 (±0.80) | 18 | 7.7 (±0.91) | 11 | 7.5 (±1.63) | |
|  | **S3** | 10 | | 7.5 (±1.72) | | 5 | | 7.6 (±0.89) | | 8 | 7.5 (±0.76) | 6 | 8.0 (±1.41) | 7 | 8.4 (±1.27) | |
|  | **S4** | 14 | | 7.5 (±0.76) | | 12 | | 7.7 (±1.07) | | 15 | 8.0 (±0.65) | 17 | 7.7 (±1.21) | 12 | 8.2 (±0.83) | |
| ***Adaptability:* In my opinion there are enough options to adapt the activities to best fit the school.** | **S1** | 13 | | 6.3 (±1.49) | | 9 | | 6.3 (±1.58) | | 12 | 7.3 (±0.75) | 4 | 7.3 (±0.50) | 12 | 6.3 (±1.15) | |
|  | **S2** | 10 | | 7.2 (±0.63) | | 6 | | 7.8 (±0.98) | | 15 | 7.3 (±1.10) | 18 | 7.6 (±1.04) | 15 | 7.4 (±0.91) | |
|  | **S3** | 10 | | 7.6 (±1.58) | | 6 | | 7.5 (±0.55) | | 8 | 7.6 (±0.92) | 5 | 8.2 (±1.10) | 9 | 8.5 (±1.00) | |
|  | **S4** | 14 | | 7.5 (±0.85) | | 15 | | 7.4 (±0.99) | | 16 | 7.7 (±0.87) | 17 | 7.7 (±1.10) | 12 | 7.9 (±0.67) | |
| ***Compatibility*: In my opinion do the activities fit within my regular work.** | **S1** | 11 | | 6.2 (±1.40) | | 9 | | 6.2 (±1.64) | | 9 | 6.8 (±1.39) | 4 | 7.0 (±1.15) | 12 | 6.1 (±2.13) | |
|  | **S2** | 10 | | 8.1 (±1.01) | | 6 | | 7.7 (±0.82) | | 15 | 7.3 (±1.22) | 17 | 7.6 (±1.12) | 13 | 7.4 (±1.19) | |
|  | **S3** | 10 | | 7.9 (±1.20) | | 6 | | 7.2 (±0.75) | | 8 | 7.8 (±1.04) | 5 | 8.6 (±1.34) | 6 | 8.6 (±0.80) | |
|  | **S4** | 14 | | 7.4 (±1.69) | | 11 | | 7.6 (±1.12) | | 16 | 7.9 (±1.06) | 14 | 8.1 (±1.07) | 12 | 8.0 (±0.95) | |
| **Support** | | | | | | | | | | | | | | | | |
| ***Support from colleagues:* In my opinion I can count on enough support from my colleagues if I need it when implementing the activities.** | **S1** | 11 | | 7.5 (±1.21) | | 9 | | 7.7 (±1.66) | | 10 | 8.5 (±0.97) | 4 | 8.5 (±0.58) | 13 | 8.2 (±1.34) | |
|  | **S2** | 10 | | 8.3 (±0.82) | | 4 | | 9.0 (±0.82) | | 15 | 8.1 (±1.36) | 18 | 8.3 (±0.96) | 11 | 9.0 (±0.89) | |
|  | **S3** | 10 | | 8.1 (±1.52) | | 4 | | 7.0 (±1.15) | | 8 | 7.8 (±1.49) | 6 | 8.2 (±1.47) | 9 | 8.9 (±1.05) | |
|  | **S4** | 13 | | 8.2 (±0.95) | | 9 | | 7.9 (±1.27) | | 16 | 8.1 (±0.72) | 15 | 8.3 (±0.98) | 12 | 8.4 (±0.90) | |
| ***Support from RPHS*: In my opinion I can count on enough support from the RPHS if I need it when implementing the activities.** | **S1** | 10 | | 6.6 (±1.17) | | 7 | | 7.1 (±1.22) | | 7 | 7.7 (±1.11) | 4 | 6.8 (±1.26) | 10 | 7.8 (±1.23) | |
|  | **S2** | 10 | | 7.6 (±1.07) | | 4 | | 8.5 (±1.29) | | 11 | 7.5 (±1.57) | 15 | 7.5 (±1.51) | 9 | 8.0 (±1.00) | |
|  | **S3** | 8 | | 7.0 (±1.69) | | 1 | | 8.0 (±.0.00) | | 7 | 7.7 (±1.38) | 1 | **5.0 (±.0.00)** | 6 | 8.5 (±0.84) | |
|  | **S4** | 10 | | 6.8 (±1.23) | | 6 | | 6.7 (±0.52) | | 13 | 7.1 (±1.71) | 13 | 7.5 (±0.88) | 9 | 7.3 (±1.32) | |
| ***Support from school coordinator:* In my opinion I can count on enough support from the school coordinator if I need it when implementing the activities.** | **S1** | 11 | | 7.5 (±1.21) | | 8 | | 8.0 (±1.20) | | 10 | 8.7 (±0.95) | 4 | 9.0 (±0.00) | 13 | 8.9 (±1.50) | |
|  | **S2** | 9 | | 8.8 (±0.83) | | 4 | | 9.5 (±1.00) | | 16 | 8.6 (±1.26) | 17 | 8.8 (±1.07) | 15 | 8.8 (±0.94) | |
|  | **S3** | 10 | | 7.3 (±1.95) | | 3 | | 8.0 (±0.00) | | 8 | 7.9 (±1.55) | 6 | 8.0 (±1.10) | 8 | 8.9 (±0.99) | |
|  | **S4** | 13 | | 8.1 (±1.32) | | 8 | | 8.3 (±1.49) | | 15 | 8.3 (±0.72) | 15 | 8.7 (±0.96) | 11 | 8.5 (±0.93) | |
| ***Support from parents:* In my opinion In my opinion I can count on enough support from parents if I need it when implementing the activities.** | **S1** | 11 | | 6.6 (±1.12) | | 7 | | 7.4 (±1.13) | | 9 | 7.7 (±1.15) | 4 | 7.5 (±0.58) | 10 | 7.4 (±0.97) | |
|  | **S2** | 10 | | 7.6 (±0.97) | | 4 | | 6.3 (±2.22) | | 16 | 7.9 (±1.06) | 18 | 7.4 (±0.92) | 14 | 6.2 (±2.01) | |
|  | **S3** | 10 | | 6.1 (±2.47) | | 2 | | 6.5 (±2.12) | | 8 | 7.0 (±1.31) | 6 | 7.7 (±1.37) | 5 | 8.4 (±0.89) | |
|  | **S4** | 13 | | 6.8 (±1.72) | | 9 | | 7.0 (±0.87) | | 15 | 7.1 (±1.46) | 14 | 7.8 (±0.67) | 12 | 8.1 (±1.28) | |
| ***Support from the sports functionary:* In my opinion I can count on enough support from the sports functionary if I need it when implementing he activities.** | **S1** | 9 | | 6.2 (±1.09) | | 7 | | 6.9 (±1.35) | | 5 | 8.2 (±1.10) | 2 | 6.5 (±2.12) | 8 | 7.9 (±1.64) | |
|  | **S2** | 10 | | 7.5 (±1.35) | | 4 | | 8.5 (±0.58) | | 13 | 8.2 (±1.21) | 15 | 7.9 (±1.19) | 7 | 8.1 (±1.77) | |
|  | **S3** | 10 | | 6.4 (±2.27) | | 2 | | 8.5 (±0.71) | | 7 | 7.7 (±1.60) | 1 | 10. (±.0.00) | 2 | 9.0 (±1.41) | |
|  | **S4** | 12 | | 7.5 (±1.00) | | 5 | | 7.4 (±0.89) | | 14 | 7.8 (±0.89) | 14 | 7.8 (±0.80) | 10 | 7.9 (±1.10) | |
| ***Support from Sodexo:* In my opinion I can count on enough support from Sodexo if I need it when implementing the activities** | **S1** | 10 | | 6.5 (±1.27) | | 7 | | 6.1 (±1.57) | | 9 | 8.0 (±0.71) | 3 | 7.7 (±0.58) | 10 | 7.1 (±1.79) | |
|  | **S2** | 10 | | 7.2 (±0.63) | | 4 | | 7.8 (±1.50) | | 15 | 7.6 (±1.18) | 19 | 7.9 (±0.88) | 13 | 7.0 (±1.53) | |
|  | **S3** | - | | - | | - | | - | | - | - | - | - | - | - | |
|  | **S4** | - | | - | | - | | - | | - | - | - | - | - | - | |
| ***Support from Maastricht University:* In my opinion I can count on enough support from UM if I need it when implementing the activities.** | **S1** | 10 | | 7.1 (±1.37) | | 6 | | **5.2 (±1.60)** | | 7 | 8.1 (±1.07) | 3 | 7.0 (±1.73) | 11 | 7.5 (±1.69) | |
|  | **S2** | 10 | | 7.6 (±0.84) | | 4 | | 8.0 (±1.41) | | 13 | 7.6 (±1.66) | 13 | 7.5 (±1.05) | 9 | 7.4 (±1.59) | |
|  | **S3** | 10 | | 6.9 (±1.97) | | 3 | | 6.7 (±1.15) | | 7 | 7.3 (±1.11) | 2 | 8.0 (±0.00) | 6 | 8.3 (±1.03) | |
|  | **S4** | 12 | | 7.7 (±0.98) | | 7 | | 6.3 (±2.50) | | 13 | 7.4 (±0.64) | 13 | 7.5 (±0.88) | 10 | 7.5 (±1.21) | |
| ***Availability of information:* I have access to all necessary information regarding the activities.** | **S1** | 11 | | 6.5 (±0.93) | | 7 | | 7.3 (±0.76) | | 9 | 7.7 (±1.32) | 3 | 7.0 (±0.00) | 11 | 6.9 (±1.30) | |
|  | **S2** | 10 | | 7.3 (±1.06) | | 6 | | 7.5 (±2.43) | | 15 | 7.7 (±0.82) | 18 | 7.6 (±0.98) | 13 | 7.8 (±0.73) | |
|  | **S3** | 9 | | 8.0 (±1.12) | | 6 | | 7.3 (±1.03) | | 8 | 7.8 (±1.28) | 6 | **5.8 (±2.79)** | 9 | 8.7 (±0.71) | |
|  | **S4** | 16 | | 7.3 (±1.39) | | 12 | | 7.3 (±1.78) | | 15 | 7.3 (±1.00) | 16 | 7.6 (±1.31) | 12 | 7.6 (±1.24) | |
| ***Support from resources/materials:* In my opinion I receive enough material support (information, materials and guidance).** | **S1** | 11 | | 6.7 (±1.49) | | 8 | | 6.9 (±1.46) | | 8 | 7.5 (±1.31) | 4 | 7.8 (±0.50) | 11 | 6.9 (±1.51) | |
|  | **S2** | 10 | | 7.9 (±0.57) | | 5 | | 6.6 (±2.07) | | 14 | 7.7 (±1.33) | 18 | 7.6 (±0.98) | 11 | 7.7 (±0.90) | |
|  | **S3** | 9 | | 7.7 (±2.00) | | 5 | | 7.6 (±1.14) | | 8 | 7.6 (±1.51) | 6 | 7.5 (±1.97) | 6 | 8.3 (±1.03) | |
|  | **S4** | 14 | | 7.8 (±0.89) | | 10 | | 7.7 (±1.06) | | 16 | 7.9 (±0.96) | 14 | 7.9 (±1.17) | 12 | 8.0 (±1.21) | |
| **Organisation: school** | | | | | | | | | | | | | | | | |
| ***School climate*: In my opinion the activities fit well within the school’s climate.** | **S1** | 13 | | 7.6 (±0.77) | | 12 | | 7.7 (±0.49) | | 14 | 8.1 (±0.83) | 4 | 8.5 (±0.58) | 15 | 7.9 (±0.83) | |
|  | **S2** | 10 | | 8.1 (±0.57) | | 6 | | 8.5 (±0.84) | | 16 | 8.4 (±1.03) | 20 | 8.4 (±1.23) | 18 | 7.9 (±1.08) | |
|  | **S3** | 10 | | 7.8 (±1.48) | | 9 | | 8.3 (±0.71) | | 8 | 8.4 (±1.19) | 6 | 9.0 (±0.89) | 9 | 8.7 (±0.87) | |
|  | **S4** | 16 | | 7.9 (±1.00) | | 17 | | 8.0 (±1.12) | | 19 | 8.2 (±1.07) | 18 | 8.3 (±0.67) | 12 | 8.5 (±1.00) | |
| ***Personnel capacity:* In my opinion there is enough personnel to implement the activities successfully.** | **S1** | 13 | | 6.4 (±1.50) | | 11 | | 6.7 (±1.35) | | 13 | 7.0 (±1.45) | 4 | 7.3 (±0.50) | 15 | 6.3 (±1.79) | |
|  | **S2** | 9 | | 6.9 (±1.36) | | 6 | | 6.3 (±2.42) | | 16 | 7.8 (±1.11) | 20 | 7.7 (±1.17) | 18 | 7.2 (±1.18) | |
|  | **S3** | 10 | | 7.5 (±2.32) | | 9 | | 8.2 (±0.83) | | 8 | 7.3 (±1.75) | 5 | 8.4 (±1.14) | 9 | 8.6 (±0.73) | |
|  | **S4** | 16 | | 7.5 (±1.46) | | 17 | | 7.6 (±1.00) | | 19 | 8.2 (±0.95) | 18 | 8.4 (±0.92) | 12 | 8.1 (±1.18) | |
| ***Financial resources:* In my opinion there are enough financial resources to perform the activities successfully.** | **S1** | 13 | | 6.9 (±1.44) | | 10 | | 7.1 (±1.60) | | 11 | 7.3 (±1.27) | 4 | 7.5 (±0.58) | 11 | 6.9 (±1.51) | |
|  | **S2** | 10 | | 7.0 (±1.33) | | 6 | | 6.3 (±2.34) | | 14 | 8.2 (±0.98) | 15 | 7.9 (±1.30) | 15 | 7.3 (±1.80) | |
|  | **S3** | 10 | | 7.8 (±1.48) | | 9 | | 8.2 (±0.83) | | 8 | 7.4 (±1.51) | 5 | 8.0 (±1.22) | 9 | 8.2 (±1.09) | |
|  | **S4** | 14 | | 7.7 (±0.83) | | 13 | | 7.3 (±1.44) | | 15 | 7.6 (±1.39) | 13 | 7.8 (±1.74) | 10 | 7.9 (±1.06) | |
| ***Available time:* In my opinion there is enough time to implement the activities successfully.** | **S1** | 13 | | 6.6 (±1.04) | | 11 | | 7.4 (±1.50) | | 15 | 7.4 (±1.06) | 4 | 6.8 (±2.63) | 15 | 7.0 (±1.41) | |
|  | **S2** | 10 | | 7.1 (±1.37) | | 6 | | 7.8 (±1.17) | | 16 | 7.3 (±2.08) | 19 | 7.6 (±1.54) | 18 | 7.6 (±0.98) | |
|  | **S3** | 10 | | 7.8 (±1.75) | | 9 | | 8.1 (±1.05) | | 8 | 7.6 (±1.51) | 6 | 7.7 (±2.25) | 9 | 8.1 (±1.17) | |
|  | **S4** | 15 | | 7.4 (±1.72) | | 16 | | 6.6 (±1.41) | | 19 | 7.8 (±1.18) | 17 | 7.6 (±1.42) | 12 | 7.9 (±1.08) | |
| ***Available materials*: In my opinion there are enough available materials and resources to implement the activities successfully.** | **S1** | 13 | | 6.8 (±1.14) | | 9 | | 7.7 (±0.71) | | 13 | 7.5 (±0.66) | 4 | 7.0 (±1.41) | 14 | 7.0 (±1.57) | |
|  | **S2** | 10 | | 6.3 (±1.70) | | 5 | | 6.8 (±1.79) | | 15 | 7.2 (±1.70) | 19 | 7.5 (±1.54) | 17 | 7.0 (±1.32) | |
|  | **S3** | 10 | | 7.5 (±1.90) | | 9 | | 8.1 (±1.17) | | 8 | 7.6 (±1.51) | 6 | 7.6 (±1.28) | 9 | 8.2 (±0.97) | |
|  | **S4** | 13 | | 7.8 (±0.73) | | 16 | | 7.8 (±1.00) | | 18 | 7.7 (±1.72) | 16 | 7.8 (±1.11) | 12 | 7.9 (±1.24) | |
| ***Communication on task responsibility:* In my opinion it is clearly communicated what each person’s tasks and esponsibilities are regarding the activities.** | **S1** | 13 | | 6.6 (±1.12) | | 9 | | 7.7 (±1.12) | | 12 | 7.4 (±1.16) | 4 | 7.3 (±0.96) | 13 | 6.8 (±1.68) | |
|  | **S2** | 9 | | 7.8 (±1.09) | | 5 | | 8.0 (±1.00) | | 15 | 7.9 (±0.80) | 20 | 7.7 (±1.53) | 18 | 7.9 (±1.18) | |
|  | **S3** | 10 | | 7.5 (±1.65) | | 8 | | 7.6 (±0.92) | | 8 | 7.1 (±1.73) | 5 | 7.6 (±1.52) | 9 | 8.4 (±0.88) | |
|  | **S4** | 16 | | 8.1 (±0.68) | | 13 | | 7.8 (±1.17) | | 17 | 7.8 (±1.01) | 16 | 7.7 (±1.13) | 12 | 7.8 (±1.42) | |
| ***Training opportunity:* In my opinion there are enough opportunities to improve my knowledge and skills regarding the activities.** | **S1** | 11 | | 6.8 (±0.61) | | 8 | | 7.0 (±1.31) | | 5 | 6.8 (±1.10) | 3 | 7.0 (±1.00) | 10 | 6.1 (±1.66) | |
|  | **S2** | 9 | | 7.9 (±0.78) | | 4 | | 7.3 (±0.50) | | 16 | 7.2 (±1.72) | 19 | 7.3 (±1.16) | 11 | 7.5 (±1.21) | |
|  | **S3** | 9 | | 7.3 (±1.87) | | 4 | | 7.0 (±1.15) | | 7 | 7.0 (±1.73) | 6 | 7.5 (±1.64) | 7 | 8.0 (±1.15) | |
|  | **S4** | 12 | | 7.7 (±0.78) | | 9 | | 6.7 (±1.22) | | 13 | 7.5 (±0.82) | 14 | 7.5 (±0.85) | 12 | 7.6 (±1.16) | |
| ***School policy:* In my opinion the activities fit well with the school’s policies.** | **S1** | 13 | | 7.8 (±0.73) | | 13 | | 8.1 (±0.76) | | 15 | 7.9 (±1.13) | 4 | 8.0 (±0.00) | 15 | 8.2 (±0.94) | |
|  | **S2** | 10 | | 8.2 (±0.79) | | 6 | | 8.7 (±0.52) | | 16 | 8.1 (±1.86) | 19 | 8.4 (±1.26) | 17 | 8.1 (±0.98) | |
|  | **S3** | 10 | | 7.8 (±1.48) | | 9 | | 8.6 (±0.73) | | 8 | 8.0 (±1.07) | 6 | 8.5 (±1.38) | 9 | 9.1 (±0.78) | |
|  | **S4** | 16 | | 8.1 (±0.77) | | 16 | | 7.9 (±1.12) | | 18 | 8.4 (±0.85) | 18 | 8.3 (±0.67) | 12 | 8.3 (±0.78) | |
| ***Informed on activity opportunities:* I am well informed regarding the choice of activities.** | **S1** | 12 | | 6.1 (±1.98) | | 10 | | 6.6 (±1.08) | | 11 | 7.3 (±2.05) | 3 | 7.0 (±1.00) | 14 | 6.7 (±2.02) | |
|  | **S2** | 10 | | 7.5 (±1.84) | | 4 | | 7.8 (±0.96) | | 14 | 7.4 (±1.50) | 20 | 7.1 (±1.68) | 14 | 7.4 (±1.39) | |
|  | **S3** | 10 | | 7.6 (±1.71) | | 8 | | 7.8 (±1.28) | | 8 | 7.5 (±1.85) | 5 | 7.8 (±1.48) | 9 | 8.8 (±1.20) | |
|  | **S4** | 16 | | 8.1 (±0.96) | | 15 | | 8.3 (±1.19) | | 19 | 8.5 (±1.07) | 17 | 7.9 (±0.83) | 12 | 8.0 (±1.10) | |
| ***Involved in decision of activities:* I am well involved in the choice of activities.** | **S1** | 12 | | **5.8 (±1.91)** | | 10 | | **5.6 (±2.22)** | | 9 | **5.0 (±2.24)** | 3 | 6.0 (±2.65) | 12 | **5.8 (±2.70)** | |
|  | **S2** | 9 | | 7.2 (±1.92) | | 6 | | 6.7 (±2.25) | | 13 | 7.0 (±1.78) | 19 | 6.4 (±1.35) | 12 | **5.8 (±1.91)** | |
|  | **S3** | 10 | | 7.2 (±2.04) | | 5 | | 6.8 (±1.10) | | 8 | 7.0 (±2.14) | 5 | 7.6 (±1.82) | 9 | 8.0 (±1.22) | |
|  | **S4** | 13 | | 6.9 (±1.89) | | 9 | | 6.1 (±2.19) | | 14 | 7.8 (±1.05) | 15 | 7.2 (±0.86) | 10 | 7.5 (±0.97) | |
| ***Subjective norm of other teachers and PE:* In my opinion other teacher and PE think it is important that I implement the activities.** | **S1** | 11 | | 7.5 (±1.13) | | 10 | | **5.4 (±2.76)** | | 8 | 8.0 (±1.20) | 2 | 7.5 (±0.71) | 13 | 7.8 (±1.46) | |
|  | **S2** | 10 | | 7.9 (±1.85) | | 5 | | 9.0 (±1.00) | | 15 | 8.1 (±1.36) | 19 | 8.3 (±1.15) | 14 | 8.7 (±0.91) | |
|  | **S3** | 9 | | 7.0 (±1.94) | | 2 | | 7.5 (±0.71) | | 7 | 8.0 (±1.29) | 6 | 8.7 (±1.21) | 6 | 8.5 (±1.52) | |
|  | **S4** | 11 | | 7.6 (±0.92) | | 6 | | 7.2 (±1.17) | | 13 | 7.9 (±0.76) | 13 | 8.0 (±0.78) | 12 | 8.2 (±0.78) | |
| ***Subjective norm from school board*: In my opinion the school board thinks it is important that I implement the activities.** | **S1** | 12 | | 7.8 (±1.19) | | 10 | | 6.1 (±3.20) | | 8 | 8.5 (±0.76) | 2 | 8.5 (±0.71) | 13 | 8.5 (±1.13) | |
|  | **S2** | 10 | | 8.1 (±1.85) | | 5 | | 9.2 (±0.84) | | 16 | 8.5 (±1.41) | 19 | 8.6 (±1.21) | 14 | 8.9 (±0.73) | |
|  | **S3** | 9 | | 7.8 (±1.48) | | 2 | | 8.5 (±0.71) | | 7 | 8.4 (±1.40) | 6 | 8.8 (±1.17) | 6 | 9.0 (±1.26) | |
|  | **S4** | 11 | | 7.8 (±0.88) | | 6 | | 7.8 (±1.17) | | 13 | 8.5 (±1.13) | 14 | 7.9 (±0.74) | 12 | 8.4 (±0.71) | |
| ***Structure/routine:* The daily routine in school is structured.** | **S1** | 14 | | 8.1 (±1.00) | | 14 | | 8.6 (±1.15) | | 16 | 8.3 (±1.00) | 6 | 8.0 (±1.26) | 16 | 8.3 (±1.29) | |
|  | **S2** | 10 | | 8.4 (±1.43) | | 7 | | 9.0 (±0.82) | | 17 | 8.8 (±0.88) | 21 | 8.6 (±0.80) | 18 | 8.5 (±0.78) | |
|  | **S3** | 10 | | 8.7 (±0.95) | | 8 | | 9.1 (±0.64) | | 8 | 8.3 (±1.28) | 6 | 8.5 (±0.84) | 9 | 9.0 (±1.00) | |
|  | **S4** | 17 | | 8.6 (±0.87) | | 19 | | 8.4 (±0.77) | | 19 | 8.5 (±0.70) | 19 | 8.8 (±0.71) | 12 | 8.8 (±0.87) | |
| **Socio-political environment** | | | | | | | | | | | | | | | | |
| ***Fit with Dutch educational policy*: In my opinion HPSF fits well within the Dutch educational policy.** | **S1** | 13 | | 7.4 (±1.12) | | 14 | | 7.9 (±1.35) | | 16 | 7.4 (±1.03) | 6 | 7.5 (±0.84) | 16 | 7.5 (±0.89) | |
|  | **S2** | 10 | | 7.9 (±1.73) | | 7 | | 8.7 (±0.95) | | 16 | 7.9 (±2.26) | 18 | 7.9 (±1.08) | 18 | 7.9 (±1.70) | |
|  | **S3** | 10 | | 7.9 (±2.33) | | 8 | | 8.4 (±1.06) | | 8 | 8.1 (±1.46) | 6 | 8.8 (±0.98) | 9 | 8.8 (±0.83) | |
|  | **S4** | 16 | | 7.7 (±1.74) | | 18 | | 7.6 (±1.20) | | 16 | 7.6 (±1.75) | 17 | 8.0 (±1.06) | 12 | 8.5 (±0.72) | |
| ***Movare supports vision of HPSF:* In my opinion Movare supports the vision of HPSF.** | **S1** | 14 | | 8.9 (±0.95) | | 14 | | 8.8 (±1.12) | | 16 | 8.3 (±1.01) | 6 | 8.0 (±0.63) | 16 | 8.3 (±0.93) | |
|  | **S2** | 10 | | 8.6 (±0.84) | | 7 | | 8.9 (±0.90) | | 16 | 8.4 (±2.19) | 21 | 9.1 (±0.83) | 18 | 8.8 (±0.92) | |
|  | **S3** | 10 | | 7.7 (±2.45) | | 8 | | 8.6 (±0.92) | | 8 | 8.0 (±1.51) | 6 | 8.8 (±0.98) | 9 | 9.0 (±1.00) | |
|  | **S4** | 17 | | 8.4 (±0.70) | | 18 | | 8.1 (±0.69) | | 17 | 8.1 (±0.70) | 19 | 8.3 (±0.93) | 12 | 8.7 (±0.89) | |
| ***Municipality supports vision of HPSF:* In my opinion the municipality supports the vision of HPSF.** | **S1** | 12 | | 8.2 (±1.11) | | 14 | | 8.0 (±1.36) | | 15 | 7.9 (±0.83) | 6 | 7.2 (±0.41) | 16 | 7.5 (±1.10) | |
|  | **S2** | 10 | | 8.0 (±1.25) | | 7 | | 8.4 (±1.27) | | 14 | 7.6 (±2.79) | 18 | 8.1 (±1.16) | 15 | 8.2 (±0.94) | |
|  | **S3** | 10 | | 7.6 (±2.17) | | 8 | | 7.5 (±1.77) | | 8 | 8.0 (±0.76) | 6 | 9.0 (±0.89) | 8 | 8.6 (±1.06) | |
|  | **S4** | 16 | | 7.7 (±1.40) | | 16 | | 7.3 (±0.95) | | 15 | 7.5 (±0.92) | 18 | 7.8 (±1.11) | 10 | 8.1 (±1.29) | |
| ***Parents support vision of HPSF:* In my opinion parents support the vision of HPSF.** | **S1** | 14 | | 7.6 (±1.04) | | 13 | | 8.1 (±1.04) | | 16 | 7.7 (±0.63) | 6 | 8.0 (±0.63) | 16 | 7.1 (±1.20) | |
|  | **S2** | 10 | | 7.8 (±1.03) | | 7 | | 8.9 (±0.69) | | 16 | 8.6 (±0.81) | 21 | 8.3 (±0.86) | 18 | 8.3 (±0.81) | |
|  | **S3** | 10 | | 7.5 (±1.65) | | 8 | | 8.1 (±0.83) | | 8 | 7.8 (±1.67) | 6 | 8.3 (±1.63) | 9 | 8.7 (±1.03) | |
|  | **S4** | 17 | | 7.3 (±1.16) | | 18 | | 7.4 (±1.24) | | 18 | 7.9 (±0.96) | 19 | 8.0 (±1.00) | 12 | 8.0 (±1.32) | |

*Bold: identified as a barrier, i.e., mean score below 6.*

**Additional file 2b. Presence of potential barriers for HPSF according to external pedagogical employees**

| **Potential barriers (1-10)** | |  | | **Nov ‘15** | | | **May ‘16** | | | **Nov ‘16** | | | | **May ‘17** | | | | **Nov ‘17** | | |
| --- | --- | --- | --- | --- | --- | --- | --- | --- | --- | --- | --- | --- | --- | --- | --- | --- | --- | --- | --- | --- |
|  |  |  |  | **N** | | **Mean (±SD)** | **N** | | **Mean (±SD)** | **N** | | **Mean (±SD)** | | **N** | | | **Mean (±SD)** | **N** | | **Mean (±SD)** |
| **Implementers: external pedagogical employees** | | | | | | | | | | | | | | | | | | | | |
| ***Attitude:* I like to implement the activities.** | **S1** | | 13 | | 8.4 (±1.12) | | 9 | 8.2 (±1.09) | | 13 | 8.6 (±0.96) | | | 6 | | 8.3 (±0.52) | | 9 | 8.7 (±0.87) | |
|  | **S2** | | 2 | | 8.0 (±0.00) | | 9 | 7.9 (±0.88) | | 11 | 7.8 (±0.75) | | | 14 | | 8.3 (±0.83) | | 13 | 8.3 (±0.63) | |
|  | **S3** | | 6 | | 8.0 (±1.26) | | 7 | 7.3 (±1.25) | | 4 | 6.9 (±2.10) | | | 4 | | 6.5 (±2.38) | | 6 | 7.6 (±0.49) | |
|  | **S4** | | 7 | | 8.6 (±1.13) | | 7 | 8.4 (±0.79) | | 8 | 8.1 (±1.13) | | | 9 | | 8.3 (±0.87) | | 7 | 8.1 (±0.90) | |
| ***Self-efficacy:* I am able to implement the activities.** | **S1** | | 13 | | 7.7 (±1.97) | | 9 | 6.8 (±1.20) | | 12 | 8.5 (±1.00) | | | 6 | | 8.0 (±0.63) | | 9 | 7.8 (±1.48) | |
|  | **S2** | | 2 | | 8.0 (±1.41) | | 9 | 7.4 (±0.98) | | 11 | 8.1 (±1.04) | | | 14 | | 7.9 (±1.33) | | 13 | 8.2 (±1.01) | |
|  | **S3** | | 6 | | 8.3 (±1.03) | | 7 | 7.9 (±0.69) | | 4 | 7.0 (±1.63) | | | 4 | | 7.0 (±1.41) | | 6 | 8.3 (±0.52) | |
|  | **S4** | | 7 | | 8.1 (±1.21) | | 7 | 8.4 (±0.79) | | 8 | 8.0 (±1.07) | | | 9 | | 8.3 (±0.87) | | 7 | 8.6 (±0.79) | |
| ***Motivation*: I am motivated to implement the activities.** | **S1** | | 13 | | 9.0 (±1.00) | | 9 | 8.1 (±1.17) | | 13 | 8.5 (±1.05) | | | 6 | | 8.2 (±0.41) | | 9 | 8.4 (±1.01) | |
|  | **S2** | | 2 | | 8.0 (±0.00) | | 9 | 7.9 (±1.01) | | 11 | 8.3 (±0.90) | | | 14 | | 8.1 (±1.21) | | 13 | 8.5 (±0.66) | |
|  | **S3** | | 6 | | 8.3 (±1.03) | | 7 | 7.4 (±1.27) | | 4 | 7.1 (±1.93) | | | 4 | | 7.3 (±1.89) | | 6 | 7.5 (±0.55) | |
|  | **S4** | | 7 | | 8.6 (±1.13) | | 7 | 8.3 (±0.76) | | 8 | 8.3 (±0.71) | | | 9 | | 8.2 (±1.09) | | 7 | 8.4 (±0.79) | |
| ***Knowledge*: I have enough knowledge to implement the activities.** | **S1** | | 13 | | 8.0 (±1.78) | | 9 | 8.1 (±1.17) | | 13 | 8.3 (±0.85) | | | 6 | | 8.2 (±0.41) | | 9 | 8.2 (±1.20) | |
|  | **S2** | | 2 | | 8.0 (±0.00) | | 9 | 7.9 (±0.73) | | 11 | 8.2 (±1.89) | | | 14 | | 8.2 (±0.98) | | 13 | 8.3 (±1.25) | |
|  | **S3** | | 6 | | 8.5 (±1.52) | | 7 | 8.0 (±0.58) | | 4 | 7.5 (±0.58) | | | 5 | | 8.6 (±0.89) | | 6 | 8.0 (±0.63) | |
|  | **S4** | | 7 | | 8.6 (±1.13) | | 7 | 7.9 (±1.07) | | 8 | 7.8 (±1.17) | | | 9 | | 8.2 (±0.44) | | 7 | 8.7 (±0.95) | |
| ***Skills:* I have enough skills to implement the activities.** | **S1** | | 13 | | 7.8 (±1.64) | | 9 | 8.1 (±1.05) | | 13 | 8.3 (±0.85) | | | 6 | | 8.2 (±0.41) | | 9 | 8.1 (±1.36) | |
|  | **S2** | | 2 | | 8.0 (±1.41) | | 9 | 7.8 (±0.83) | | 11 | 8.4 (±0.50) | | | 15 | | 8.3 (±0.82) | | 13 | 8.3 (±1.18) | |
|  | **S3** | | 6 | | 8.5 (±1.52) | | 7 | 7.6 (±1.27) | | 4 | 8.0 (±0.82) | | | 5 | | 8.6 (±0.89) | | 6 | 7.8 (±0.99) | |
|  | **S4** | | 7 | | 8.1 (±1.46) | | 7 | 7.9 (±1.07) | | 8 | 8.0 (±1.20) | | | 9 | | 8.1 (±0.63) | | 7 | 8.7 (±0.95) | |
| ***Modelling behaviour*: I try to give the good example to children concerning healthy dietary and PA behaviours.** | **S1** | | 13 | | 8.8 (±1.30) | | 9 | 8.8 (±0.97) | | 13 | 9.0 (±0.91) | | | 7 | | 9.0 (±0.82) | | 10 | 8.0 (±1.56) | |
|  | **S2** | | 2 | | 8.5 (±0.71) | | 9 | 7.4 (±0.73) | | 10 | 7.9 (±0.74) | | | 16 | | 8.0 (±1.10) | | 12 | 8.4 (±0.79) | |
|  | **S3** | | 6 | | 7.3 (±1.37) | | 7 | 7.4 (±1.27) | | 4 | 7.0 (±0.82) | | | 5 | | 8.0 (±1.58) | | 6 | 7.5 (±1.05) | |
|  | **S4** | | 7 | | 7.7 (±0.49) | | 7 | 8.0 (±1.00) | | 8 | 8.0 (±0.93) | | | 9 | | 8.4 (±0.88) | | 7 | 7.6 (±0.53) | |
| ***Satisfaction:* In my opinion the children like the activities.** | **S1** | | 12 | | 7.7 (±1.56) | | 9 | 7.8 (±1.09) | | 13 | 7.7 (±1.32) | | | 6 | | 8.0 (±0.89) | | 9 | 7.1 (±1.83) | |
|  | **S2** | | 2 | | 7.5 (±0.71) | | 9 | 7.2 (±0.67) | | 11 | 8.0 (±0.89) | | | 15 | | 7.1 (±1.54) | | 12 | 7.8 (±0.75) | |
|  | **S3** | | 6 | | 7.7 (±1.37) | | 7 | **5.9 (±1.77)** | | 4 | **5.1 (±1.18)** | | | 5 | | **5.4 (±1.95)** | | 6 | 6.8 (±0.98) | |
|  | **S4** | | 7 | | 7.6 (±0.53) | | 7 | 7.0 (±1.00) | | 8 | 7.5 (±1.20) | | | 9 | | 7.8 (±0.97) | | 7 | 8.0 (±0.58) | |
| ***Cooperation:* In my opinion the children cooperate well with the activities.** | **S1** | | 12 | | 7.1 (±1.73) | | 9 | 7.0 (±1.73) | | 13 | 7.4 (±1.39) | | | 6 | | 6.7 (±1.37) | | 9 | 6.8 (±1.64) | |
|  | **S2** | | 2 | | 8.0 (±1.41) | | 8 | 6.9 (±0.83) | | 11 | 7.3 (±1.01) | | | 16 | | 7.0 (±1.19) | | 13 | 7.4 (±1.19) | |
|  | **S3** | | 6 | | 7.2 (±0.75) | | 7 | **5.9 (±1.77)** | | 4 | **4.8 (±0.96)** | | | 5 | | **5.0 (±2.12)** | | 6 | 6.5 (±0.84) | |
|  | **S4** | | 7 | | 7.0 (±2.45) | | 7 | 7.0 (±0.00) | | 8 | 7.5 (±0.76) | | | 9 | | 7.6 (±1.22) | | 7 | 8.0 (±0.58) | |
| ***Perceived outcome importance*: In my opinion children have healthier dietary and PA behaviours due to the activities.** | **S1** | | 13 | | 8.7 (±0.95) | | 9 | 7.7 (±1.41) | | 13 | 8.3 (±1.18) | | | 6 | | 7.7 (±1.03) | | 9 | 7.2 (±1.39) | |
|  | **S2** | | 2 | | 8.5 (±2.12) | | 9 | 7.6 (±1.13) | | 11 | 7.3 (±1.49) | | | 16 | | 7.6 (±1.26) | | 13 | 7.5 (±1.61) | |
|  | **S3** | | 6 | | **5.7 (±0.82)** | | 7 | **4.9 (±2.79)** | | 3 | **3.0 (±1.73)** | | | 5 | | **4.8 (±2.05)** | | 5 | **5.6 (±1.52)** | |
|  | **S4** | | 7 | | **5.3 (±1.70)** | | 7 | **5.9 (±1.46)** | | 8 | 6.9 (±1.08) | | | 9 | | 6.3 (±1.12) | | 7 | 6.6 (±1.27) | |
| ***Personal advantage:* I see also other advantages of the activities.** | **S1** | | 13 | | 8.1 (±1.26) | | 9 | 7.4 (±1.50) | | 13 | 8.0 (±1.15) | | | 6 | | 7.8 (±0.75) | | 9 | 7.2 (±1.64) | |
|  | **S2** | | 2 | | 7.5 (±0.71) | | 9 | 6.6 (±1.33) | | 11 | 7.3 (±1.10) | | | 16 | | 6.9 (±1.71) | | 13 | 7.5 (±1.27) | |
|  | **S3** | | 6 | | **5.8 (±1.17)** | | 7 | **5.7 (±2.14)** | | 3 | 6.3 (±1.53) | | | 5 | | **5.4 (±2.51)** | | 6 | 6.0 (±1.41) | |
|  | **S4** | | 7 | | 7.4 (±1.40) | | 7 | 7.3 (±0.76) | | 8 | 7.5 (±0.76) | | | 9 | | 8.0 (±1.00) | | 7 | 7.0 (±1.41) | |
| ***Identification with organization:* I feel involved with the school I am working at.** | **S1** | | 13 | | 7.9 (±1.44) | | 9 | 8.7 (±1.12) | | 13 | 9.0 (±1.00) | | | 7 | | 8.6 (±1.27) | | 10 | 8.9 (±1.20) | |
|  | **S2** | | 2 | | 7.5 (±0.71) | | 9 | 6.8 (±0.67) | | 10 | 7.6 (±0.97) | | | 16 | | 8.0 (±0.94) | | 13 | 8.6 (±0.51) | |
|  | **S3** | | 6 | | **5.5 (±1.64)** | | 7 | 6.4 (±1.72) | | 4 | 6.5 (±2.08) | | | 5 | | 6.2 (±1.30) | | 7 | 6.6 (±0.98) | |
|  | **S4** | | 7 | | 7.4 (±1.40) | | 7 | 7.4 (±0.79) | | 8 | 7.8 (±1.17) | | | 9 | | 7.9 (±1.36) | | 7 | 7.4 (±1.13) | |
| ***Perceived outcome feasibility*: In my opinion it is important for children to have healthier dietary and PA behaviours.** | **S1** | | 13 | | 9.2 (±0.80) | | 9 | 8.9 (±0.93) | | 13 | 9.5 (±0.66) | | | 7 | | 9.1 (±0.69) | | 10 | 9.3 (±0.95) | |
|  | **S2** | | 2 | | 8.0 (±0.00) | | 9 | 8.4 (±0.86) | | 10 | 8.4 (±0.84) | | | 16 | | 8.3 (±0.93) | | 13 | 8.5 (±0.97) | |
|  | **S3** | | 6 | | 8.7 (±1.21) | | 7 | 9.1 (±0.90) | | 4 | 8.8 (±0.96) | | | 5 | | 8.8 (±0.84) | | 7 | 8.1 (±0.73) | |
|  | **S4** | | 7 | | 8.6 (±1.13) | | 7 | 8.6 (±0.85) | | 8 | 8.9 (±0.83) | | | 9 | | 9.0 (±0.71) | | 7 | 8.4 (±0.79) | |
| ***Perceived task responsibility:* In my opinion it is one of the school’s responsibilities to encourage children to have healthier dietary and PA behaviours.** | **S1** | | 13 | | 8.1 (±0.86) | | 9 | 7.1 (±1.05) | | 13 | 7.5 (±1.56) | | | 7 | | 7.9 (±1.07) | | 10 | 7.9 (±1.10) | |
|  | **S2** | | 2 | | 8.0 (±0.00) | | 9 | 7.4 (±0.53) | | 10 | 7.1 (±1.42) | | | 16 | | 6.8 (±1.65) | | 12 | 7.4 (±1.62) | |
|  | **S3** | | 6 | | 7.5 (±1.38) | | 7 | 9.0 (±0.82) | | 4 | **5.8 (±0.96)** | | | 5 | | 6.8 (±0.84) | | 7 | 7.0 (±1.15) | |
|  | **S4** | | 7 | | 6.3 (±1.50) | | 7 | 7.6 (±1.27) | | 8 | 6.0 (±2.56) | | | 9 | | 8.0 (±1.22) | | 7 | 7.3 (±1.38) | |
| **Innovation: HPSF** | | | | | | | | | | | | | | | | | | | | |
| *****Required time:* In my opinion it costs a lot of time to implement the activities.** | **S1** | | 10 | | **5.9 (±2.02)** | | 8 | **5.9 (±2.80)** | | 13 | **5.6 (±2.50)** | | 6 | | **5.5 (±2.81)** | | | 9 | **5.2 (±3.03)** | |
|  | **S2** | | 2 | | **5.0 (±2.83)** | | 9 | **5.0 (±1.32)** | | 10 | 7.0 (±1.70) | | 15 | | 6.5 (±1.55) | | | 13 | **4.2 (±2.01)** | |
|  | **S3** | | 5 | | 8.4 (±1.52) | | 7 | **3.1 (±2.67)** | | 4 | 8.0 (±2.16) | | 5 | | 6.4 (±2.19) | | | 6 | 7.2 (±1.83) | |
|  | **S4** | | 7 | | **4.6 (±1.81)** | | 6 | 6.3 (±2.07) | | 8 | 7.5 (±1.77) | | 9 | | 6.9 (±1.54) | | | 7 | 6.6 (±2.51) | |
| *****Complexity:* In my opinion the activities are complex to implement.** | **S1** | | 13 | | 7.0 (±2.77) | | 9 | 6.2 (±1.48) | | 13 | 7.1 (±2.29) | | 6 | | 6.5 (±2.43) | | | 9 | 6.2 (±2.99) | |
|  | **S2** | | 2 | | 6.0 (±2.83) | | 8 | **4.3 (±2.22)** | | 11 | 8.7 (±0.65) | | 15 | | 8.5 (±0.83) | | | 13 | 7.9 (±1.75) | |
|  | **S3** | | 6 | | 9.3 (±0.52) | | 7 | **2.3 (±2.14)** | | 3 | 6.7 (±2.52) | | 5 | | 9.0 (±0.71) | | | 5 | 7.8 (±1.30) | |
|  | **S4** | | 7 | | 7.9 (±2.04) | | 7 | **4.0 (±2.31)** | | 8 | 8.0 (±2.00) | | 9 | | 8.4 (±2.13) | | | 7 | 8.1 (±3.18) | |
| ***Relevance:* In my opinion the activities are suitable for children.** | **S1** | | 13 | | 8.2 (±2.01) | | 9 | 7.2 (±1.50) | | 13 | 7.3 (±2.14) | | 6 | | 7.7 (±1.97) | | | 9 | 8.0 (±0.71) | |
|  | **S2** | | 2 | | 7.5 (±0.71) | | 9 | 6.8 (±0.83) | | 11 | 7.8 (±0.60) | | 15 | | 7.8 (±1.21) | | | 13 | 8.2 (±0.73) | |
|  | **S3** | | 6 | | 7.8 (±1.17) | | 7 | 6.4 (±2.64) | | 4 | **4.9 (±1.65)** | | 5 | | 6.8 (±1.30) | | | 6 | 7.5 (±0.55) | |
|  | **S4** | | 7 | | 6.7 (±2.06) | | 7 | 6.4 (±2.23) | | 8 | 7.5 (±1.20) | | 9 | | 7.8 (±1.20) | | | 6 | 7.7 (±1.03) | |
| ***Observability*: In my opinion the effects of the activities are clearly visible.** | **S1** | | 8 | | 7.4 (±1.88) | | 9 | 6.9 (±1.83) | | 13 | 7.5 (±1.33) | | 6 | | 6.8 (±1.17) | | | 9 | 6.8 (±1.48) | |
|  | **S2** | | 2 | | 6.5 (±0.71) | | 9 | 6.3 (±1.41) | | 11 | 6.1 (±1.14) | | 15 | | 6.7 (±1.05) | | | 13 | 6.8 (±1.01) | |
|  | **S3** | | 6 | | **4.2 (±2.32)** | | 7 | **5.1 (±2.91)** | | 2 | **3.5 (±2.12)** | | 5 | | **3.8 (±1.10)** | | | 7 | **5.9 (±1.21)** | |
|  | **S4** | | 7 | | 6.0 (±1.00) | | 7 | 6.4 (±0.53) | | 8 | 6.9 (±1.36) | | 8 | | 7.5 (±0.93) | | | 7 | 6.7 (±1.25) | |
| ***Flexibility*: In my opinion the activities are flexible enough to work with.** | **S1** | | 13 | | 8.0 (±1.08) | | 9 | 7.8 (±1.17) | | 13 | 7.8 (±1.21) | | 6 | | 7.7 (±0.82) | | | 9 | 7.7 (±1.00) | |
|  | **S2** | | 2 | | 7.5 (±0.71) | | 9 | 7.1 (±1.27) | | 11 | 7.5 (±0.82) | | 16 | | 7.5 (±1.55) | | | 13 | 7.2 (±1.63) | |
|  | **S3** | | 6 | | **5.7 (±1.21)** | | 7 | 6.1 (±2.04) | | 4 | **4.8 (±2.22)** | | 4 | | **4.5 (±1.91)** | | | 6 | 7.2 (±0.41) | |
|  | **S4** | | 7 | | 7.4 (±1.40) | | 7 | 7.4 (±1.27) | | 8 | 7.3 (±1.04) | | 9 | | 8.4 (±0.70) | | | 7 | 7.4 (±1.13) | |
| ***Adaptability:* In my opinion there are enough options to adapt the activities to best fit the school.** | **S1** | | 12 | | 7.5 (±1.45) | | 9 | 7.6 (±1.51) | | 13 | 7.4 (±1.26) | | 6 | | 7.8 (±0.75) | | | 9 | 7.6 (±1.13) | |
|  | **S2** | | 2 | | 7.5 (±2.12) | | 9 | **5.8 (±1.62)** | | 11 | 7.8 (±0.60) | | 16 | | 7.6 (±1.50) | | | 11 | 7.3 (±1.79) | |
|  | **S3** | | 6 | | **5.8 (±1.33)** | | 7 | **5.4 (±2.82)** | | 3 | **3.7 (±2.08)** | | 5 | | **4.6 (±1.14)** | | | 6 | 7.0 (±0.63) | |
|  | **S4** | | 7 | | 7.4 (±0.98) | | 7 | 6.9 (±1.22) | | 8 | 7.0 (±1.07) | | 9 | | 7.6 (±1.13) | | | 7 | 7.1 (±1.46) | |
| ***Compatibility*: In my opinion do the activities fit within my regular work.** | **S1** | | 11 | | 8.4 (±1.69) | | 9 | 7.9 (±1.36) | | 13 | 8.2 (±0.90) | | 6 | | 8.2 (±0.41) | | | 9 | 8.1 (±1.05) | |
|  | **S2** | | 2 | | 8.0 (±1.41) | | 9 | 6.8 (±1.50) | | 11 | 7.7 (±0.65) | | 13 | | 7.8 (±1.07) | | | 13 | 7.3 (±2.02) | |
|  | **S3** | | 6 | | 6.7 (±1.97) | | 7 | 6.4 (±2.15) | | 3 | 6.7 (±1.15) | | 4 | | **5.8 (±3.30)** | | | 6 | 7.3 (±0.52) | |
|  | **S4** | | 7 | | 8.0 (±1.29) | | 7 | 8.0 (±1.00) | | 8 | 7.6 (±0.52) | | 9 | | 7.9 (±0.78) | | | 7 | 7.6 (±1.13) | |
| **Support** | | | | | | | | | | | | | | | | | | | | |
| ***Support from colleagues:* In my opinion I can count on enough support from my colleagues if I need it when implementing the activities.** | **S1** | | 12 | | 7.8 (±1.85) | | 9 | 8.0 (±1.12) | | 13 | 8.5 (±0.66) | | 6 | | 8.3 (±0.82) | | | 9 | 8.7 (±1.00) | |
|  | **S2** | | 2 | | **5.5 (±0.71)** | | 9 | 7.6 (±1.13) | | 11 | 7.5 (±0.69) | | 15 | | 8.0 (±0.90) | | | 13 | 8.9 (±0.76) | |
|  | **S3** | | 6 | | 7.2 (±1.94) | | 7 | 7.7 (±0.49) | | 4 | 8.0 (±1.15) | | 5 | | 7.4 (±1.67) | | | 6 | 7.0 (±0.89) | |
|  | **S4** | | 7 | | 8.0 (±1.63) | | 7 | 8.1 (±1.07) | | 8 | 9.0 (±0.93) | | 9 | | 8.3 (±1.50) | | | 7 | 8.3 (±0.95) | |
| ***Support from RPHS*: In my opinion I can count on enough support from the RPHS if I need it when implementing the activities.** | **S1** | | 8 | | 6.3 (±1.58) | | 9 | **5.2 (±2.17)** | | 10 | 7.4 (±2.17) | | 5 | | 7.4 (±1.14) | | | 4 | 7.5 (±1.00) | |
|  | **S2** | | 2 | | **3.8 (±1.77)** | | 4 | **5.8 (±1.26)** | | 4 | **5.8 (±2.22)** | | 5 | | 7.0 (±1.73) | | | 4 | 6.3 (±1.50) | |
|  | **S3** | | 5 | | **4.2 (±2.05)** | | 2 | **5.0 (±2.83)** | | 2 | **3.5 (±0.71)** | | 0 | | - | | | 3 | **4.7 (±1.53)** | |
|  | **S4** | | 5 | | 6.0 (±1.58) | | 7 | **3.3 (±2.75)** | | 5 | **4.8 (±2.28)** | | 6 | | 6.8 (±2.86) | | | 3 | 7.0 (±1.73) | |
| ***Support from school coordinator:* In my opinion I can count on enough support from the school coordinator if I need it when implementing the activities.** | **S1** | | 13 | | 7.7 (±1.39) | | 9 | 8.1 (±1.05) | | 12 | 8.2 (±1.27) | | 6 | | 8.2 (±0.75) | | | 8 | 8.8 (±1.16) | |
|  | **S2** | | 2 | | **5.0 (±4.24)** | | 9 | 7.1 (±1.36) | | 9 | 7.6 (±1.24) | | 12 | | 8.0 (±1.01) | | | 11 | 8.9 (±0.83) | |
|  | **S3** | | 6 | | 6.7 (±1.03) | | 7 | 6.9 (±0.90) | | 2 | 6.5 (±0.71) | | 5 | | **5.4 (±3.13)** | | | 6 | 7.2 (±0.98) | |
|  | **S4** | | 6 | | 7.3 (±1.75) | | 7 | 6.9 (±2.35) | | 6 | 8.2 (±3.13) | | 8 | | 9.0 (±0.93) | | | 6 | 8.5 (±0.84) | |
| ***Support from parents:* In my opinion In my opinion I can count on enough support from parents if I need it when implementing the activities.** | **S1** | | 8 | | 6.5 (±0.93) | | 9 | 6.1 (±2.20) | | 8 | 6.1 (±2.53) | | 5 | | 6.8 (±1.48) | | | 4 | 7.8 (±0.50) | |
|  | **S2** | | 2 | | **4.5 (±3.54)** | | 8 | **4.8 (±2.48)** | | 7 | 6.0 (±2.71) | | 5 | | 6.6 (±1.34) | | | 7 | **5.0 (±2.24)** | |
|  | **S3** | | 5 | | **4.0 (±1.87)** | | 5 | **3.2 (±2.68)** | | 2 | **3.5 (±2.12)** | | 3 | | **3.7 (±2.08)** | | | 2 | **3.0 (±2.83)** | |
|  | **S4** | | 6 | | 7.0 (±1.90) | | 6 | 7.8 (±1.60) | | 6 | 7.3 (±1.97) | | 6 | | 7.5 (±1.64) | | | 6 | 8.5 (±0.84) | |
| ***Support from the sports functionary:* In my opinion I can count on enough support from the sports functionary if I need it when implementing the activities.** | **S1** | | 9 | | 7.2 (±1.30) | | 8 | 7.0 (±1.07) | | 10 | 7.5 (±2.55) | | 4 | | 7.3 (±1.71) | | | 5 | 8.2 (±1.10) | |
|  | **S2** | | 2 | | 7.0 (±1.41) | | 5 | 6.8 (±1.30) | | 6 | 6.7 (±1.51) | | 5 | | 8.2 (±0.84) | | | 6 | 8.3 (±1.03) | |
|  | **S3** | | 4 | | **4.5 (±2.38)** | | 7 | **5.3 (±2.50)** | | 3 | **4.7 (±2.52)** | | 3 | | **5.7 (±0.58)** | | | 2 | 6.5 (±0.71) | |
|  | **S4** | | 5 | | 6.2 (±1.10) | | 7 | 6.1 (±2.85) | | 7 | 7.9 (±1.95) | | 6 | | 7.7 (±2.42) | | | 6 | 6.8 (±1.47) | |
| ***Support from Sodexo:* In my opinion I can count on enough support from Sodexo if I need it when implementing the activities** | **S1** | | 7 | | 6.7 (±0.76) | | 8 | 6.8 (±1.28) | | 9 | 8.6 (±1.13) | | 6 | | 8.0 (±0.89) | | | 7 | 8.4 (±0.98) | |
|  | **S2** | | 2 | | 6.5 (±2.12) | | 4 | **4.5 (±2.52)** | | 6 | 6.8 (±2.93) | | 6 | | 6.7 (±2.94) | | | 7 | 6.7 (±0.49) | |
|  | **S3** | | - | | - | | - | - | | - | - | | - | | - | | | - | - | |
|  | **S4** | | - | | - | | - | - | | - | - | | - | | - | | | - | - | |
| ***Support from Maastricht University:* In my opinion I can count on enough support from UM if I need it when implementing the activities.** | **S1** | | 7 | | 7.0 (±1.15) | | 9 | 6.7 (±1.32) | | 9 | 7.6 (±2.23) | | 6 | | 7.0 (±1.26) | | | 5 | 8.0 (±0.71) | |
|  | **S2** | | 2 | | **4.0 (±1.41)** | | 5 | **5.0 (±2.45)** | | 5 | 6.0 (±1.73) | | 4 | | 7.5 (±1.00) | | | 7 | 6.0 (±1.00) | |
|  | **S3** | | 3 | | **5.3 (±0.58)** | | 5 | **5.4 (±1.67)** | | 1 | **3.0 (±0.00)** | | 1 | | **2.0 (±0.00)** | | | 2 | **4.0 (±1.41)** | |
|  | **S4** | | 5 | | **5.8 (±1.30)** | | 7 | **3.6 (±2.70)** | | 5 | **4.8 (±2.86)** | | 3 | | 8.3 (±1.53) | | | 2 | 6.0 (±1.41) | |
| ***Availability of information:* I have access to all necessary information regarding the activities.** | **S1** | | 13 | | 8.1 (±1.55) | | 9 | 7.8 (±1.20) | | 13 | 8.0 (±1.22) | | 6 | | 8.5 (±0.55) | | | 9 | 8.1 (±0.60) | |
|  | **S2** | | 2 | | 7.0 (±0.00) | | 9 | 7.1 (±0.60) | | 11 | 8.1 (±0.70) | | 15 | | 7.8 (±1.41) | | | 13 | 7.7 (±0.88) | |
|  | **S3** | | 6 | | 6.0 (±2.37) | | 7 | 6.0 (±2.31) | | 4 | **5.8 (±2.50)** | | 5 | | **3.8 (±2.28)** | | | 6 | 6.8 (±0.98) | |
|  | **S4** | | 7 | | 7.4 (±0.79) | | 7 | 7.7 (±1.60) | | 8 | 8.4 (±0.52) | | 9 | | 8.1 (±0.60) | | | 7 | 8.3 (±0.76) | |
| ***Support from resources/materials:* In my opinion I receive enough material support (information, materials and guidance).** | **S1** | | 13 | | 7.2 (±2.41) | | 9 | 7.0 (±1.50) | | 13 | 7.9 (±0.95) | | 6 | | 7.0 (±0.89) | | | 9 | 7.4 (±1.33) | |
|  | **S2** | | 2 | | 7.0 (±0.00) | | 9 | 6.6 (±1.01) | | 8 | 7.9 (±0.83) | | 15 | | 7.1 (±1.25) | | | 13 | 8.0 (±1.15) | |
|  | **S3** | | 6 | | 6.3 (±1.21) | | 7 | **5.9 (±1.95)** | | 4 | **5.5 (±1.91)** | | 5 | | **3.2 (±1.48)** | | | 6 | 6.7 (±0.82) | |
|  | **S4** | | 7 | | 7.6 (±1.40) | | 7 | 7.9 (±1.57) | | 8 | 8.4 (±0.92) | | 9 | | 8.6 (±0.73) | | | 7 | 8.4 (±0.79) | |
| **Organisation: school** | | | | | | | | | | | | | | | | | | | | |
| ***School climate*: In my opinion the activities fit well within the school’s climate.** | **S1** | | 13 | | 8.2 (±0.80) | | 9 | 7.7 (±1.32) | | 13 | 8.2 (±0.80) | | 6 | | 8.0 (±0.63) | | | 9 | 8.0 (±1.12) | |
|  | **S2** | | 2 | | 7.5 (±0.71) | | 8 | 6.8 (±0.89) | | 11 | 7.5 (±0.93) | | 16 | | 7.9 (±0.96) | | | 13 | 8.0 (±0.58) | |
|  | **S3** | | 4 | | **5.0 (±2.58)** | | 7 | 6.0 (±2.45) | | 4 | **5.5 (±2.89)** | | 5 | | **4.6 (±2.61)** | | | 6 | 6.8 (±0.41) | |
|  | **S4** | | 7 | | 7.9 (±0.90) | | 7 | 7.0 (±1.91) | | 8 | 8.1 (±0.99) | | 9 | | 7.9 (±1.36) | | | 7 | 7.9 (±0.38) | |
| ***Personnel capacity:* In my opinion there is enough personnel to implement the activities successfully.** | **S1** | | 12 | | 7.1 (±2.22) | | 9 | 6.6 (±1.74) | | 13 | 7.5 (±1.61) | | 6 | | 6.7 (±1.21) | | | 10 | 7.0 (±1.94) | |
|  | **S2** | | 2 | | **4.0 (±2.83)** | | 9 | 6.1 (±1.27) | | 11 | 6.5 (±1.57) | | 16 | | 6.9 (±1.39) | | | 13 | 7.3 (±1.32) | |
|  | **S3** | | 6 | | **5.5 (±1.87)** | | 7 | 6.4 (±2.88) | | 4 | 6.0 (±1.83) | | 5 | | **5.4 (±3.36)** | | | 6 | 6.7 (±0.82) | |
|  | **S4** | | 7 | | **5.7 (±2.43)** | | 7 | 7.6 (±1.40) | | 8 | 7.9 (±1.13) | | 9 | | 7.9 (±1.45) | | | 7 | 7.7 (±0.49) | |
| ***Financial resources:* In my opinion there are enough financial resources to perform the activities successfully.** | **S1** | | 11 | | 8.2 (±1.25) | | 7 | 7.7 (±1.25) | | 10 | 8.4 (±0.97) | | 6 | | 7.0 (±1.10) | | | 5 | 7.6 (±1.52) | |
|  | **S2** | | 1 | | 7.0 (±0.00) | | 8 | **4.8 (±1.58)** | | 7 | 7.0 (±1.15) | | 15 | | 7.1 (±1.19) | | | 11 | 8.0 (±0.77) | |
|  | **S3** | | 4 | | 6.5 (±1.00) | | 6 | 6.8 (±1.94) | | 3 | **4.7 (±2.52)** | | 3 | | **4.3 (±0.58)** | | | 4 | 6.8 (±0.96) | |
|  | **S4** | | 7 | | 7.4 (±1.40) | | 7 | 7.6 (±1.31) | | 8 | 8.0 (±1.07) | | 9 | | 8.1 (±0.60) | | | 7 | 8.0 (±0.58) | |
| ***Available time:* In my opinion there is enough time to implement the activities successfully.** | **S1** | | 12 | | 8.0 (±0.95) | | 9 | 7.1 (±1.76) | | 13 | 7.5 (±1.05) | | 6 | | 7.7 (±1.03) | | | 9 | 6.6 (±1.74) | |
|  | **S2** | | 2 | | **5.0 (±1.41)** | | 9 | 6.4 (±0.60) | | 11 | 7.4 (±1.12) | | 16 | | 6.9 (±1.63) | | | 13 | 6.6 (±1.85) | |
|  | **S3** | | 6 | | **4.5 (±2.07)** | | 7 | **5.7 (±1.80)** | | 4 | **5.0 (±2.16)** | | 5 | | 6.4 (±1.14) | | | 6 | 7.0 (±0.00) | |
|  | **S4** | | 7 | | 6.3 (±1.60) | | 7 | 6.0 (±1.41) | | 8 | 7.4 (±1.51) | | 9 | | 7.6 (±0.53) | | | 7 | 7.6 (±0.53) | |
| ***Available materials*: In my opinion there are enough available materials and resources to implement the activities successfully.** | **S1** | | 11 | | 7.5 (±1.92) | | 9 | 8.0 (±1.00) | | 13 | 7.4 (±1.12) | | 6 | | 6.8 (±0.98) | | | 9 | 6.3 (±2.00) | |
|  | **S2** | | 2 | | 7.0 (±1.41) | | 9 | **5.2 (±1.92)** | | 11 | 7.7 (±0.90) | | 16 | | 7.1 (±1.06) | | | 13 | 7.9 (±1.04) | |
|  | **S3** | | 6 | | **5.8 (±1.17)** | | 7 | 6.3 (±2.29) | | 4 | **5.3 (±2.36)** | | 5 | | **3.6 (±1.52)** | | | 6 | 6.8 (±0.41) | |
|  | **S4** | | 7 | | 7.7 (±1.11) | | 7 | 7.8 (±1.35) | | 8 | 8.0 (±1.07) | | 9 | | 7.9 (±1.17) | | | 7 | 8.0 (±0.82) | |
| ***Communication on task responsibility:* In my opinion it is clearly communicated what each person’s tasks and responsibilities are regarding the activities.** | **S1** | | 13 | | 7.8 (±1.74) | | 9 | 7.9 (±1.27) | | 13 | 7.8 (±1.21) | | 6 | | 8.3 (±0.52) | | | 9 | 7.7 (±1.94) | |
|  | **S2** | | 2 | | **5.5 (±0.71)** | | 9 | 6.9 (±1.27) | | 11 | 7.8 (±0.98) | | 16 | | 7.6 (±1.26) | | | 13 | 8.5 (±1.13) | |
|  | **S3** | | 6 | | 6.8 (±1.17) | | 7 | 6.7 (±2.06) | | 4 | **5.8 (±2.06)** | | 5 | | 6.0 (±2.83) | | | 6 | 6.3 (±1.51) | |
|  | **S4** | | 7 | | 7.7 (±0.76) | | 7 | 7.9 (±1.07) | | 8 | 8.5 (±0.93) | | 9 | | 8.2 (±0.71) | | | 7 | 7.6 (±1.13) | |
| ***Training opportunity:* In my opinion there are enough opportunities to improve my knowledge and skills regarding the activities.** | **S1** | | 13 | | 7.4 (±1.94) | | 9 | 7.3 (±1.22) | | 13 | 8.2 (±0.73) | | 6 | | 8.0 (±0.63) | | | 8 | 8.0 (±1.20) | |
|  | **S2** | | 2 | | **4.5 (±3.54)** | | 9 | 6.7 (±0.71) | | 11 | 6.9 (±1.87) | | 15 | | 7.0 (±1.46) | | | 13 | 7.9 (±0.86) | |
|  | **S3** | | 6 | | **5.8 (±1.33)** | | 6 | 7.0 (±1.26) | | 3 | 6.7 (±2.52) | | 5 | | **5.6 (±2.70)** | | | 6 | 7.2 (±0.75) | |
|  | **S4** | | 7 | | 7.7 (±1.11) | | 7 | 7.2 (±1.63) | | 8 | 8.0 (±2.20) | | 9 | | 8.4 (±0.73) | | | 7 | 8.0 (±0.58) | |
| ***School policy:* In my opinion the activities fit well with the school’s policies.** | **S1** | | 13 | | 8.7 (±0.78) | | 9 | 8.0 (±1.12) | | 13 | 8.5 (±0.66) | | 6 | | 8.0 (±0.63) | | | 9 | 8.1 (±0.93) | |
|  | **S2** | | 2 | | 7.5 (±0.71) | | 8 | 6.6 (±0.52) | | 11 | 7.6 (±0.81) | | 16 | | 7.9 (±0.93) | | | 12 | 8.4 (±1.00) | |
|  | **S3** | | 5 | | **5.2 (±1.92)** | | 6 | 6.3 (±1.21) | | 3 | **5.3 (±2.52)** | | 5 | | **5.4 (±2.70)** | | | 6 | 7.0 (±0.00) | |
|  | **S4** | | 7 | | 8.0 (±1.15) | | 7 | 7.9 (±1.22) | | 8 | 8.1 (±0.83) | | 9 | | 7.9 (±0.33) | | | 7 | 7.6 (±1.13) | |
| ***Informed on activity opportunities:* I am well informed regarding the choice of activities.** | **S1** | | 13 | | 8.2 (±1.14) | | 9 | 7.6 (±1.13) | | 13 | 7.8 (±1.24) | | 6 | | 7.8 (±0.75) | | | 9 | 8.2 (±0.83) | |
|  | **S2** | | 2 | | **5.0 (±2.83)** | | 9 | 6.4 (±1.33) | | 10 | 6.8 (±1.23) | | 16 | | 6.9 (±1.63) | | | 12 | 8.1 (±0.67) | |
|  | **S3** | | 6 | | 6.3 (±1.51) | | 7 | 6.4 (±2.07) | | 4 | **4.9 (±1.65)** | | 5 | | **4.8 (±2.39)** | | | 7 | 6.7 (±0.95) | |
|  | **S4** | | 7 | | 6.9 (±0.90) | | 6 | 7.7 (±0.52) | | 8 | 8.4 (±1.19) | | 9 | | 8.1 (±0.60) | | | 7 | 8.0 (±0.58) | |
| ***Involved in decision of activities:* I am well involved in the choice of activities.** | **S1** | | 13 | | 8.0 (±1.41) | | 9 | 6.9 (±1.76) | | 13 | 7.6 (±1.19) | | 6 | | 7.5 (±1.38) | | | 9 | 6.8 (±1.80) | |
|  | **S2** | | 2 | | **5.5 (±3.54)** | | 9 | 6.6 (±0.86) | | 11 | 6.4 (±1.43) | | 16 | | 6.1 (±2.08) | | | 13 | 8.2 (±0.69) | |
|  | **S3** | | 6 | | **5.7 (±1.21)** | | 7 | 6.7 (±2.43) | | 4 | 6.3 (±2.63) | | 5 | | **5.4 (±2.61)** | | | 6 | 7.3 (±0.82) | |
|  | **S4** | | 7 | | 7.1 (±0.69) | | 7 | 6.7 (±1.70) | | 8 | 8.1 (±1.57) | | 9 | | 8.2 (±1.20) | | | 7 | 8.1 (±0.38) | |
| ***Subjective norm of other teachers and PE:* In my opinion other teacher and PE think it is important that I implement the activities.** | **S1** | | 13 | | 8.6 (±1.04) | | 8 | 7.8 (±1.51) | | 13 | 8.5 (±0.88) | | 6 | | 8.5 (±0.84) | | | 9 | 8.6 (±1.13) | |
|  | **S2** | | 2 | | 7.5 (±0.71) | | 8 | 7.5 (±0.53) | | 10 | 7.4 (±0.70) | | 15 | | 7.7 (±0.88) | | | 13 | 8.5 (±0.78) | |
|  | **S3** | | 6 | | **5.5 (±0.84)** | | 6 | 6.8 (±1.47) | | 4 | 6.3 (±2.99) | | 5 | | **5.6 (±2.97)** | | | 6 | 7.5 (±1.22) | |
|  | **S4** | | 7 | | 7.7 (±0.49) | | 7 | 7.4 (±0.98) | | 8 | 8.3 (±1.04) | | 9 | | 8.0 (±1.32) | | | 7 | 7.4 (±1.13) | |
| ***Subjective norm from school board*: In my opinion the school board thinks it is important that I implement the activities.** | **S1** | | 12 | | 8.7 (±1.07) | | 8 | 8.3 (±1.17) | | 13 | 8.7 (±0.75) | | 6 | | 8.7 (±0.52) | | | 9 | 8.7 (±1.00) | |
|  | **S2** | | 2 | | 8.0 (±1.41) | | 9 | 7.5 (±0.50) | | 10 | 7.6 (±0.70) | | 15 | | 7.9 (±0.88) | | | 12 | 8.4 (±0.79) | |
|  | **S3** | | 6 | | 6.2 (±1.94) | | 7 | 7.3 (±1.98) | | 4 | 6.3 (±2.99) | | 5 | | **5.6 (±2.97)** | | | 6 | 7.3 (±1.51) | |
|  | **S4** | | 7 | | 7.7 (±0.49) | | 7 | 7.9 (±0.38) | | 8 | 8.4 (±0.92) | | 9 | | 7.9 (±1.36) | | | 7 | 7.9 (±1.57) | |
| ***Structure/routine:* The daily routine in school is structured.** | **S1** | | 11 | | 8.1 (±1.30) | | 9 | 8.1 (±0.93) | | 12 | 8.2 (±1.12) | | 7 | | 7.9 (±1.22) | | | 10 | 8.3 (±1.25) | |
|  | **S2** | | 2 | | 8.5 (±0.71) | | 9 | 6.5 (±1.12) | | 10 | 7.9 (±0.74) | | 16 | | 7.9 (±1.32) | | | 13 | 8.1 (±1.12) | |
|  | **S3** | | 5 | | 7.2 (±1.92) | | 6 | 6.7 (±1.86) | | 4 | 7.0 (±1.41) | | 5 | | **5.0 (±0.71)** | | | 6 | 6.7 (±1.03) | |
|  | **S4** | | 7 | | 7.7 (±0.76) | | 7 | 7.4 (±0.79) | | 8 | 8.5 (±0.93) | | 9 | | 8.2 (±1.56) | | | 7 | 7.3 (±1.11) | |
| **Socio-political environment** | | | | | | | | | | | | | | | | | | | | |
| ***Fit with Dutch educational policy*: In my opinion HPSF fits well within the Dutch educational policy.** | **S1** | | 12 | | 8.1 (±1.00) | | 9 | 8.3 (±1.15) | | 13 | 8.5 (±0.97) | | 7 | | 8.4 (±0.79) | | | 10 | 8.1 (±1.20) | |
|  | **S2** | | 1 | | 7.0 (±0.00) | | 7 | 7.0 (±0.82) | | 8 | 8.1 (±0.64) | | 15 | | 7.8 (±1.05) | | | 10 | 7.7 (±1.16) | |
|  | **S3** | | 4 | | **4.5 (±2.89)** | | 6 | 6.7 (±1.63) | | 2 | **5.5 (±0.71)** | | 4 | | **5.5 (±0.58)** | | | 6 | 6.7 (±0.52) | |
|  | **S4** | | 6 | | 7.7 (±1.37) | | 6 | 7.5 (±0.84) | | 8 | 7.4 (±1.06) | | 9 | | 7.9 (±0.78) | | | 7 | 7.1 (±1.07) | |
| ***Movare supports vision of HPSF:* In my opinion Movare supports the vision of HPSF.** | **S1** | | 12 | | 8.7 (±1.30) | | 9 | 8.2 (±1.39) | | 11 | 8.7 (±1.10) | | 7 | | 8.4 (±1.27) | | | 8 | 8.6 (±1.19) | |
|  | **S2** | | 2 | | 8.5 (±0.71) | | 7 | 7.6 (±1.13) | | 9 | 7.1 (±2.15) | | 13 | | 8.0 (±0.91) | | | 10 | 8.3 (±1.16) | |
|  | **S3** | | 4 | | **5.8 (±2.99)** | | 6 | 6.2 (±2.04) | | 3 | 6.7 (±2.08) | | 3 | | 6.7 (±2.89) | | | 5 | 6.6 (±0.55) | |
|  | **S4** | | 7 | | 8.3 (±0.95) | | 7 | 8.0 (±1.00) | | 7 | 8.1 (±0.38) | | 8 | | 8.3 (±0.59) | | | 7 | 7.3 (±1.25) | |
| ***Municipality supports vision of HPSF:* In my opinion the municipality supports the vision of HPSF.** | **S1** | | 12 | | 8.8 (±1.03) | | 9 | 7.9 (±1.27) | | 10 | 8.4 (±1.26) | | 6 | | 8.7 (±0.82) | | | 8 | 8.1 (±1.25) | |
|  | **S2** | | 1 | | 9.0 (±0.00) | | 4 | 6.8 (±1.71) | | 9 | 7.4 (±1.13) | | 13 | | 7.5 (±0.88) | | | 9 | 8.1 (±1.17) | |
|  | **S3** | | 5 | | 7.8 (±2.17) | | 7 | 7.1 (±2.04) | | 3 | 6.0 (±1.00) | | 4 | | 6.3 (±0.96) | | | 5 | 6.6 (±0.55) | |
|  | **S4** | | 7 | | 8.3 (±0.95) | | 7 | 7.0 (±1.15) | | 8 | 7.5 (±1.20) | | 7 | | 8.0 (±0.58) | | | 7 | 7.6 (±0.53) | |
| ***Parents support vision of HPSF:* In my opinion parents support the vision of HPSF.** | **S1** | | 11 | | 8.3 (±1.56) | | 9 | 7.6 (±1.42) | | 13 | 7.1 (±1.75) | | 7 | | 7.4 (±1.40) | | | 9 | 7.7 (±1.50) | |
|  | **S2** | | 2 | | 8.5 (±0.71) | | 4 | 8.0 (±0.82) | | 8 | 7.8 (±0.71) | | 14 | | 7.5 (±0.76) | | | 11 | 7.5 (±1.04) | |
|  | **S3** | | 5 | | **5.8 (±1.30)** | | 6 | 6.2 (±2.32) | | 2 | **5.0 (±0.00)** | | 3 | | **4.7 (±0.58)** | | | 3 | **5.7 (±0.58)** | |
|  | **S4** | | 7 | | 7.7 (±0.95) | | 7 | 7.0 (±1.15) | | 8 | 7.3 (±1.22) | | 8 | | 8.0 (±0.93) | | | 7 | 7.0 (±1.41) | |

*Bold: identified as a barrier, i.e., mean score below 6.*
