# Supplementary material for: Process evaluation of the healthy primary School of the Future: the key learning points
Source: BMC Public Health. 2019 Jun 6;19:698. doi: 10.1186/s12889-019-6947-2 (PMC6554901; doi:10.1186/s12889-019-6947-2)
Supplement: Supplementary file 3 — Perceived important factors for success of HPSF. Description: Perception of teachers and external pedagogical employees regarding important factors for success (DOCX 19 kb) [file 12889_2019_6947_MOESM3_ESM.docx]

**Additional file 3. Perceived important factors for success of HPSF**

**Additional file 3a. Perception of teachers: important factors for success***

|  | **Nov ‘15** | **May ‘16** | **Nov ‘16** | **Nov ‘17** |
| --- | --- | --- | --- | --- |
| **S1** | **N teachers^a^ = 14** | **N teachers = 14** | **N teachers = 16** | **N teachers = 16** |
|  | **Total N barriers^b^ = 49** | **Total N barriers = 53** | **Total N barriers = 66** | **Total N barriers = 34** |
|  | 1. Support and involvement teachers (n=11) 2. Clear communication (n=9) 3. Support and involvement parents (n=8) | 1. Efforts and motivation of teachers and external PE (n=9) 2. Support and involvement parents (n=7) 3. Sufficient facilities: money (n=3), personnel (n=3), materials (n=2) | 1. Support and involvement parents (n=9) 2. Variation in activities (n=5) 3. Contact and collaboration (n=5) | 1. Support and involvement parents (n=7) 2. Supervision TSO (n=5) 3. Active activities (n=4) |
| **S2** | **N teachers = 10** | **N teachers = 7** | **N teachers = 18** | **N teachers = 18** |
|  | **Total N barriers = 40** | **Total N barriers = 30** | **Total N barriers = 73** | **Total N barriers = 35** |
|  | 1. Motivation staff (n=6) 2. Regular evaluation, and adaptation when needed (n=4) 3. Sufficient facilities: space (n=4), money (n=3), personnel (n=2) | 1. Support, devotion of all people (n=5) 2. Relation external PE with teacher (n=3) 3. Sufficient facilities: money (n=2), time (n=1), materials (n=1), space (n=1), personnel (n=1) | 1. Support and involvement parents (n=7) 2. Collaboration (n=6) 3. Plan for future (n=3) | 1. Structure (n=6) 2. Motivation and enthusiasm of all people (n=4) 3. Sufficient facilities: personnel (n=2), money (n=2) |
| **S3** | **N teachers = 10** | **N teachers = 9** | **N teachers = 8** | **N teachers = 9** |
|  | **Total N barriers = 49** | **Total N barriers = 29** | **Total N barriers = 33** | **Total N barriers = 15** |
|  | 1. Support and involvement parents (n=7) 2. More fun, less stress for all children (n=6) 3. Sufficient facilities: time (n=2), money (n=2), space (n=2), materials (n=2), personnel (n=1), | 1. Quality PE (n=4) 2. Communication (n=3) 3. Sufficient facilities: money (n=2), materials (n=2), time (n=1), space (n=1), personnel (n=1) | 1. Involvement of all people (n=4) 2. Clear communication (n=3) 3. Sufficient facilities: personnel (n=2), money (n=1), materials (n=1), time (n=1) | 1. Informing people (n=3) 2. Collaboration (n=2) 3. Sufficient facilities: money (n=2), personnel (n=2) |
| **S4** | **N teachers = 17** | **N teachers = 19** | **N teachers = 19** | **N teachers = 12** |
|  | **Total N barriers = 62** | **Total N barriers = 81** | **Total N barriers = 80** | **Total N barriers = 24** |
|  | 1. Support and involvement parents (n=6) 2. Sufficient facilities: personnel (n=6), money (n=5), materials (n=5), space (n=4) 3. Good organisation (n=5) | 1. Sufficient facilities: money (n=12), personnel (n=7), materials (n=3), time (n=2), space (n=2) 2. Support and involvement parents (n=6) 3. Support and involvement team (n=6) | 1. Motivation, enthusiasm of all people (n=12) 2. Sufficient facilities: money (n=12), personnel (n=10), materials (n=7), space (n=3), time (n=2) 3. Structure and rules (n=3) | 1. Sufficient facilities: money (n=4), personnel (n=2) 2. Coordination (n=2) 3. Structure (n=2) |

** not asked in the open questions of the questionnaire in May ’17*

*^a^ Number of teachers who filled out the questionnaire*

*^b^ Number of barriers mentioned in total*

**Additional file 3b. Perception of external pedagogical employees: important factors for success***

|  | **Nov ‘15** | **May ‘16** | **Nov ‘16** | **Nov ‘17** |
| --- | --- | --- | --- | --- |
| **S1** | **N external PE^a^ = 13** | **N external PE = 9** | **N external PE = 13** | **N external PE = 10** |
|  | **Total N barriers^b^ = 57** | **Total N barriers = 24** | **Total N barriers = 50** | **Total N barriers = 13** |
|  | 1. Collaboration between all involved people (n=9) 2. Support and involvement parents (n=8) 3. Efforts of teachers and external PE (n=5) | 1. Collaboration and efforts of teachers and external PE (n=4) 2. Enough volunteers (n=3) 3. Structure and reliability (n=3) | 1. Collaboration and communication (n=8) 2. Motivation of all people involved (n=7) 3. Sufficient variation in activities (n=4) | 1. Collaboration (n=3) 2. Quality of lunch (n=3) 3. Support and involvement parents (n=2) |
| **S2** | **N external PE = 2** | **N external PE = 9** | **N external PE = 11** | **N external PE = 13** |
|  | **Total N barriers = 9** | **Total N barriers = 35** | **Total N barriers = 28** | **Total N barriers = 18** |
|  | 1. Sufficient variation in activities (n=2) 2. Sufficient facilities: materials (n=1), space (n=1) 3. Enthusiasm among children and staff (n=1) | 1. Shared goal and ideas (n=7) 2. Guidelines for implementation (n=6) 3. Regular evaluation (n=4) | 1. Collaboration (n=4) 2. Good atmosphere (n=4) 3. Structure (n=3) | 1. Motivation and enthusiasm of all involved people (n=5) 2. Collaboration between external PE and teachers (n=3) 3. Coordination (n=3) |
| **S3** | **N external PE = 6** | **N external PE = 7** | **N external PE = 4** | **N external PE = 7** |
|  | **Total N barriers = 11** | **Total N barriers = 26** | **Total N barriers = 17** | **Total N barriers = 14** |
|  | 1. Sufficient variation in activities (n=3) 2. Creating more PA in the school (n=2) 3. Sufficient facilities: time (n=1), personnel (n=1), materials (n=1), space (n=1) | 1. Creating more PA in the school (n=4) 2. Collaboration (n=3) 3. Sufficient facilities: time (n=1), materials (n=1) | 1. Communication between external PE and teacher (n=6) 2. Efforts of external PE (n=2) 3. Quality of activities (n=2) | 1. Sufficient information on PA and nutrition (n=4) 2. Involvement children (n=2) 3. Sufficient facilities: time (n=1), money (n=1) |
| **S4** | **N external PE = 7** | **N external PE = 7** | **N external PE = 8** | **N external PE = 7** |
|  | **Total N barriers = 30** | **Total N barriers = 27** | **Total N barriers = 30** | **Total N barriers = 7** |
|  | 1. Support and involvement teachers and external PE (n=5) 2. Sufficient variation in activities (n=4) 3. Clear communication (n=3) | 1. Challenging activities n=3) 2. Efforts and motivation of teachers and external PE (n=2) 3. Collaboration (n=2) | 1. Quality of activities (n=5) 2. Doing it together (n=3) 3. Motivation of and fun for children (n=3) | 1. Collaboration (n=3) 2. Communication (n=1) 3. Encouragement of children (n=1) |

** not asked in the open questions of the questionnaire in May ’17*

*^a^ Number of external PE who filled out the questionnaire*

*^b^ Number of barriers mentioned in total*
